# Tuning the thermostability of GHG gels by salts at different positions on the Hofmeister scale

**DOI:** 10.1038/s41598-024-65145-7

**Published:** 2024-06-26

**Authors:** Nichole S. O’Neill, Nicolas J. Alvarez, Reinhard Schweitzer-Stenner

**Affiliations:** 1 Department of Chemistry, Drexel University, Philadelphia, PA 19104 USA; 2 Department of Chemical and Biological Engineering, Drexel University, Philadelphia, PA 19104 USA

**Keywords:** Supramolecular assembly, Bioinspired materials

## Abstract

The influence of Hofmeister cations (NH_4_^+^, Na^+^, Mg^2+^) and anions (H_2_PO_4_^−^, CH_3_COO^−^, Cl^−^_,_ NO_3_^−^) on the thermostability of a GHG hydrogel was investigated. The combined results of UV circular dichroism (UVCD) and Small Amplitude Oscillatory Shear Rheology experiments reveal that the addition of salt reduces the stability of the gel phase and the underlying fibrils. In line with the cationic Hofmeister hierarchy, the chaotropic Mg^2+^ ions caused the greatest thermal destabilization of the gel phase with the gel → sol transition temperature *T*_*gs*_ value lowered by 10 °C. In the absence of salt, the gel → sol transition probed by the storage modulus and microscopy is biphasic. In the presence of salt, it becomes monophasic. Contrary to expectations the presence of Hofmeister anions leads to a nearly identical reduction of the gel → sol transition temperatures. However, UVCD spectra suggest that they affect the ππ-stacking between imidazole groups to a different extent. We relate the absence of ion specificity regarding the solubility of fibrils (probed by UVCD) to the observed enthalpy-entropy compensation of the dissolution process. Our results combined show how CD spectroscopy and rheology combined yields a more nuanced picture of the processes underlying the gel → sol transition.

## Introduction

**Figure 1:**
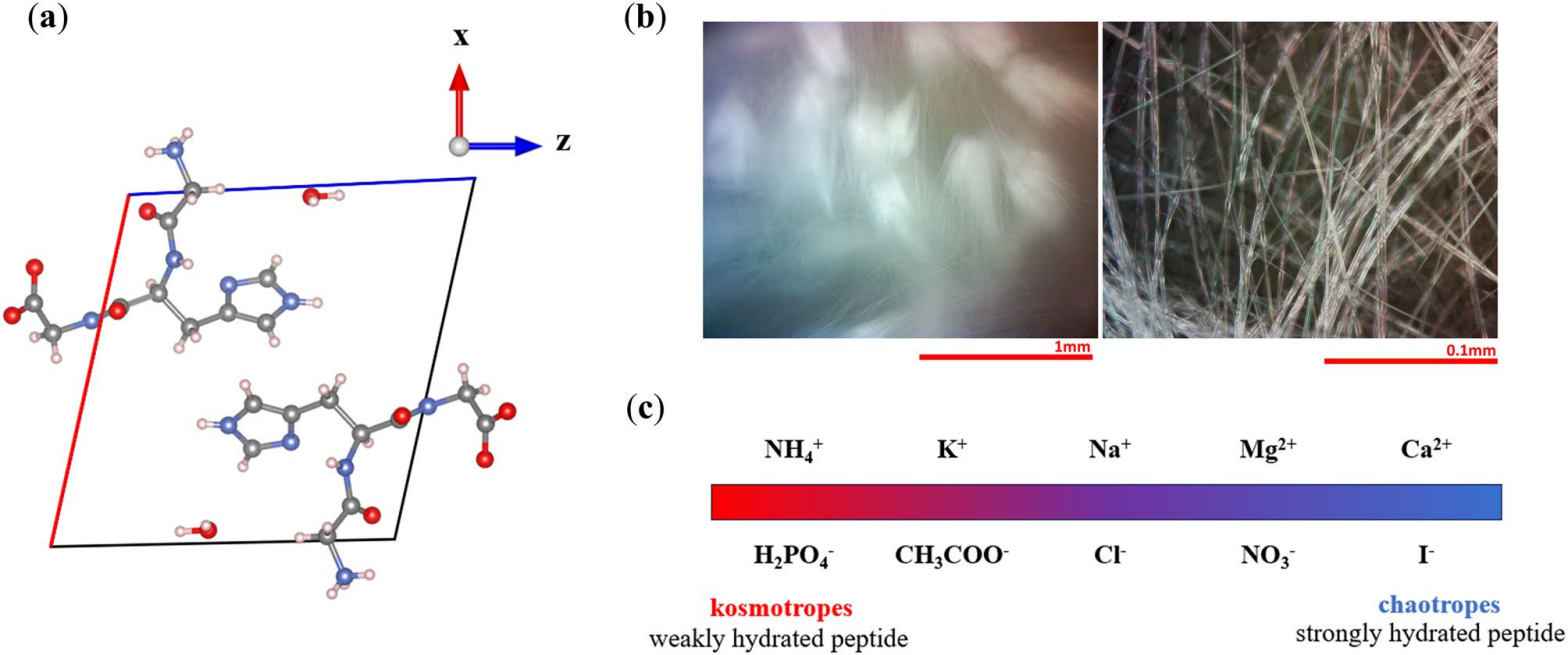
(**a**) Unit cell of GHG fibrils have *P*2_1_ symmetry. (**b**) Microscopy images of a GHG/water gel made with a 0.1 M sodium acetate solution were taken with the indicated resolution using a bright field microscope. (**c**) Hoffmeister series is divided between kosmotropes and chaotropes, which are often used to predict the “salting-out” and “salting-in” effects, respectively.

Over the last 5 years unblocked GxG peptides with aromatic guest residues were identified as ultrafast gelators in water with gel points in the centimolar and sub-molar range^1^. Recently, our work on GxG gelators focused on the self-assembly and gelation of the unblocked tripeptide Gly-His-Gly (GHG) in water (unit cell shown in Fig. 1a)^2,3^. The underlying sample spanning network contains crystalline fibrils with an exceptionally large aspect ratio, which grow from a limited number of rather large dense condensates (Fig. 1b). The entanglement of these fibrils leads to a high storage modulus that can be tuned in a range from 10^4^ to 10^6^ Pa by varying the respective monomer concentration between 50 and 300 mM and the pH of the solution between 5.5 and 6.5^3^. Our interest in this particular GxG hydrogel is based on the expectation that it could be a potential candidate for drug storage and delivery due to the controlled assembly in a narrow mildly acidic pH range. The phase diagram of a pH-switch based GHG gel clearly revealed that the self-assembly of GHG is triggered by the deprotonation of the imidazole side chain^2^. Alternatively, the gelation of zwitterionic GHG can be initiated by annealing (heat switch) at neutral pH which involves the dissolution of the peptide at 80–90 °C and the subsequent self-assembly into gel-supporting fibrils upon cooling to room temperature. The nanostructure of the fibrils revealed a plethora of intermolecular interactions that stabilize a polyproline II—like conformation in the *P*2_1_ unit cell of the crystalline fibrils (H-bonds, salt-bridges between terminal NH_3_^+^ and COO^−^ groups, and ππ-stacking), which is distinct from the usual β-sheet structure of peptide fibrils^4^.

In order to explore the usability of GHG gels for any biomedical application the stability of the gel phase has to be determined regarding external parameters like temperature and solvent composition. The knowledge of the gel softening point and the gel → sol transition temperature is important for assessing its usability at physiological temperatures and for identifying the underlying forces that stabilize or destabilize the gel phase. It is therefore astonishing that systematic studies of the thermodynamics which underlies the gelation of short and ultrashort peptides are in short supply (see Ref.^1^ for more details). Of similar importance is a detailed understanding of the influence of electrolytes on the stability of gels and structure of the underlying network^5–8^. In this context one has to recall Franz Hofmeister’s methodical study of how the presence of salt affect the stability of proteins^9^. The sequence of salts classified as the Hofmeister series divides ions into “salting-out” ions (*kosmotropes*) and “salting-in” ions (*chaotropes*) (Fig. 1c). According to Hofmeister ions affect the structure and dynamics of water with kosmotropes being “structure makers” and chaotropes being “structure breakers” of ordered water molecules beyond the first hydration shell. Structure makers would thus take water from the hydration shell of the solute. Nowadays, this view is considered as too simplistic because it overestimates the influence of ions on the structure of water in the bulk and does not take into account various direct interactions between ions and solute. For peptides and proteins this includes binding to functional peptide groups (NH and CO) and to ionized side chains^6^. For cations, interactions with carboxylate groups (ion pairing) are prominent. Generally, the actual solute–solvent interactions are often very complex in that they involve a delicate balance existing between water-water, water-ion, water-solute, and cation–anion interactions^10,11^.

There is actually a vast number of studies of how Hofmeister salts affect the formation of supramolecular structures of peptides and proteins with a strong emphasis on amyloid formation^12^. The stability of peptide gels can be affected by salts that vary the peptide’s solubility (e.g. by the screening of charges) as well as the structure of the underlying fibrils and thus the strength of the gel phase^5^. The latter has recently been demonstrated for the very hydrophobic ultrashort peptides Fmoc-VL and Fmoc-LL where different Hofmeister anions of sodium were found to significantly affect the fibril structure, the network of the gel phase, the thermal stability and the storage modulus^13^. These changes reflect the hierarchy of the Hofmeister series for anions. While Hofmeister anions are generally more effective than the respective cations to elicit ion-specific effects, divalent cations such as Ca^2+^ and Mg^2+^ have been identified as facilitators of functionalized dipeptides^7^.

The work described in this paper focused on exploring the thermal stability of GHG gels in the absence and presence of chaotropic (Mg^2+^, NO_3_^−^) and kosmotropic ions (NH_4_^+^, H_2_PO_4_^−^, CH_3_COO^−^). Recent rheological experiments indicated that the GHG gel is significantly more thermally stable than the gel phase of other GxG peptides (GAG in water/ethanol, GWG, GYG and GFG in water)^14^. We quantitatively assess the thermal stability of GHG gels by using rheology and UVCD spectroscopy. As recently demonstrated for GAG gels in water/ethanol mixtures the two techniques provide complementary information in that rheology probes the strength of a gel while UVCD reflects the internal structure of the underlying fibrils^15^. The combination of the two techniques allowed us to determine the gel → sol transition and the corresponding softening temperature as well as the dissolution temperature of the underlying fibrils. The combined results of our UVCD and rheological experiments and microscope images revealed a rather complex picture, where the Hofmeister scale constitutes only one dimension of a multi-dimensional parameter space. While the obtained results might reflect specific properties of GHG and potentially other GxG gels, the concepts governing this investigation should be applicable to other peptide gelators.

## Results

### Microscopy

**Figure 2:**
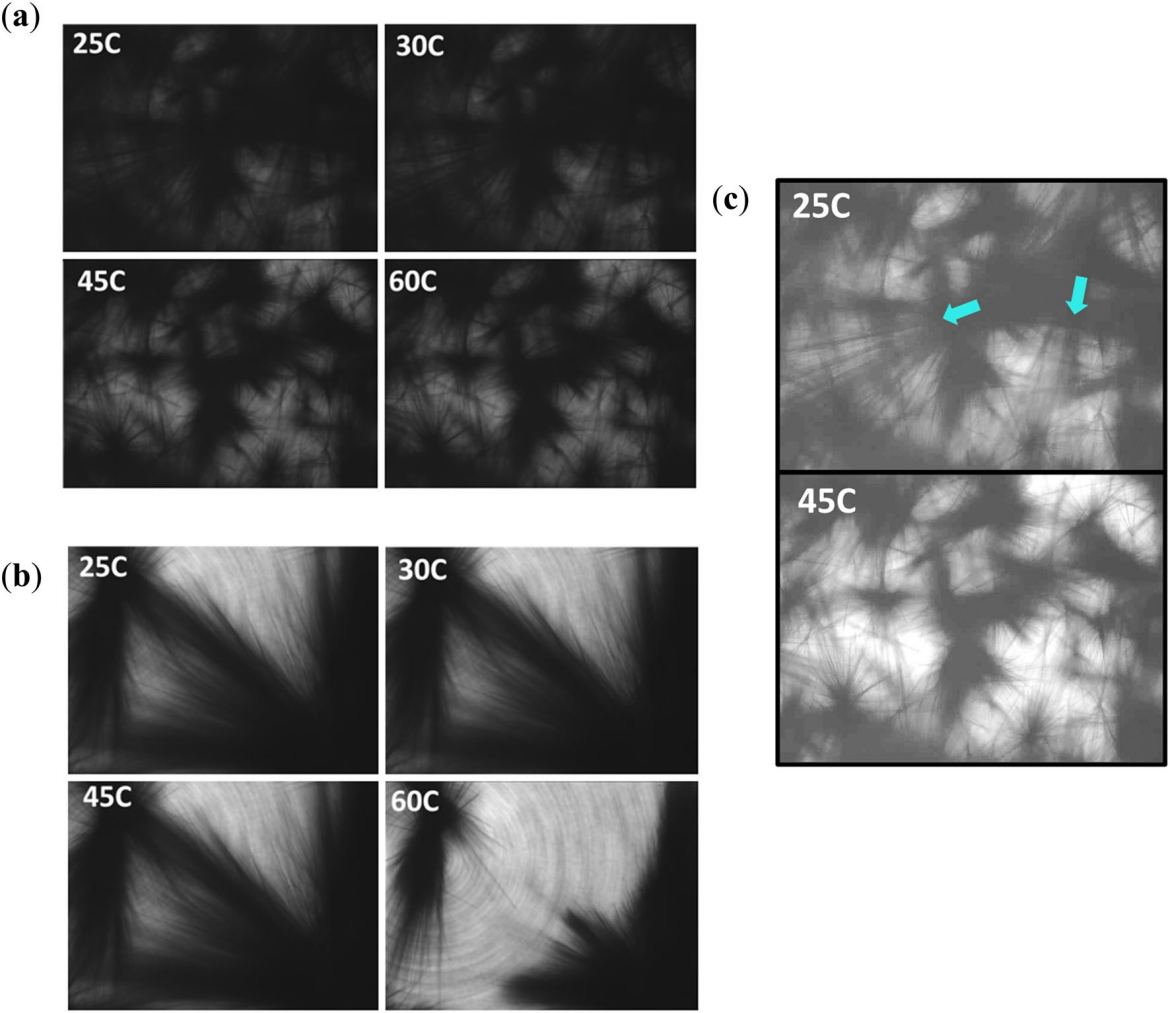
Microscopic images of GHG gel → sol transitions. Snapshots of the gel → sol process recorded on the DHR-3 show a larger number of nucleation sites in the (**a**) ‘no salt’ gel compared to the (**b**) NaCl gel. The fibrils are more homogeneous in the presence of salt with larger voids compared to the no salt gel. Images were taken using a Nikon objective with a 4 × magnification. Images in 2a were edited using brightness and contract. (**c**) Longer fibrils can be seen spanning the network in the background of smaller aggregate clusters. These longer fibrils appear to disappear first, being nearly eliminated in the 45 °C image. The blue arrows point in the direction of the fibrils, while the light blue lines run parallel to the fibrils.

To obtain a representative picture of GHG gelation we recorded optical images of the formation (Supplementary Figs. S1, S2) and melting process (Fig. 2) of 80 mM GHG gels prepared in the absence and presence of NaCl. The images were taken using the Modular Microscope Accessory for the Discovery Hybrid Rheometer (DHR-3). They show how bushels of long fibrils grow from droplet-like spots in all directions. Generally, these spots have diameters in 10^–5^ m range. We assign them to droplets formed by liquid–liquid demixing of the peptide-water mixture^16,17^. The peptide concentration in these droplets is considerably higher than it is in the mixed sample. Such a local accumulation of peptides and proteins can facilitate self-assembly into fibrils^17–20^. The comparatively large size of the droplets is a consequence of the initial peptide concentration which is more than an order of magnitude higher than the concentrations generally needed for the self-assembly of ultrashort peptides.

Figure 2 shows images of GHG gels formed in the absence and presence of NaCl. For the former they reveal two types of fibrils that are describable as long and short fibrils relative to one another. The longer ones dissolve first as the temperature increases from 25 to 45 °C, which leads to the small voids between the smaller clusters (Fig. 2c). The shorter fibrils disappear at temperatures above 70 °C. The images of the gel formed in the presence of salt have significant voids in comparison to the no salt gel with what appears to be a lower number of droplets that facilitate the formation of a more homogeneous set of fibril clusters. An increase of the voids volume only appears above 45 °C, where the direction of fibril dissolution occurs from the outer most point of the fibril inward (towards the center).

### SAOS rheology

**Figure 3:**
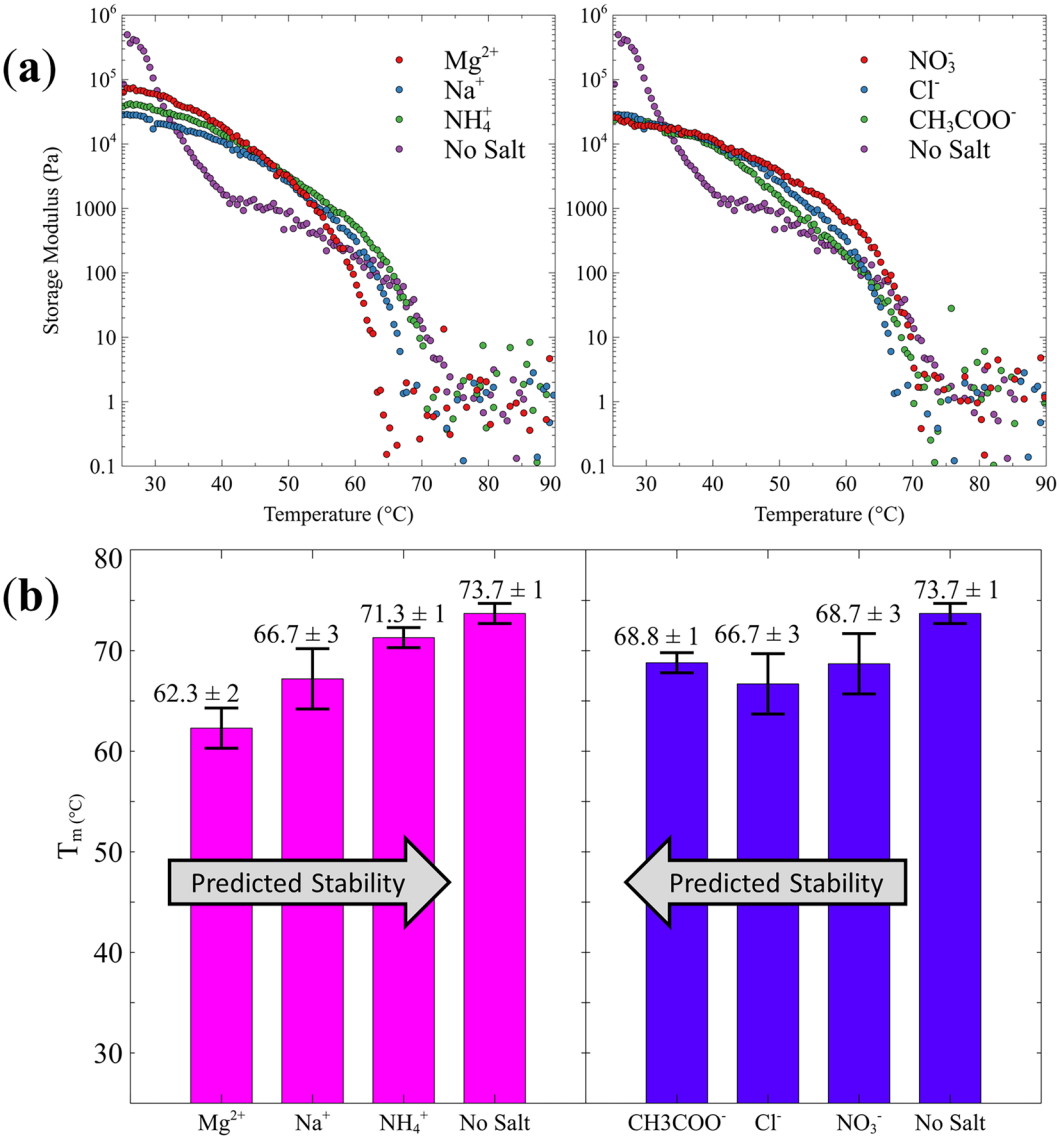
Rheological analysis of the GHG gel → sol transition. (**a**) Storage modulus G′ of GHG in the absence and presence of the indicated cations (left) and anions (right) plotted as a function of temperature. Red, blue, and green series are the chaotropes, kosmotropes, and neutral ions in both plots, respectively. (**b**) Graphic representation of the gel → sol transition temperatures of a GHG gel phase formed in the absence and the presence of the indicated salts. The salt concentration was 100 mM. Cationic and anionic effects are visualized in the left and right figure, respectively.

The thermal stability of the GHG gels were probed using SAOS. For each sample, moduli were measured as a function of time at a given temperature to ensure that a steady state was reached prior. In the gel phase, G′ exceeds G″ and G* is constant over a large range of frequencies (0.01 ≤ ω, rad/s ≤ 100)^21^. The temperature dependent storage moduli depicted in Fig. 3a are grouped by the investigated cation (NH_4_^+^, Na^+^, Mg^2+^) and anion series (CH_3_COO^−^, Cl^−^_,_ NO_3_^−^), respectively. The softening curves for Mg^2+^ and NH_4_^+^ were both measured in triplicate with high reproducibility and small errors for *T*_*sg*_ (Supplementary Fig. S3). All samples formed a gel network, as indicated by storage moduli in the 10^4^ Pa range at room temperature (see Supplementary Fig. S4 for a complete comparison of G′ and G″ values). However, these values are all lower than G′ measured in the absence of salt at room temperature (~ 10^5^ Pa). For all samples, the data indicate a continuous loss of the connectivity between fibrils with increasing temperature. In the absence of salts, the gel → sol transition is biphasic while it is monophasic in the presence of salt. It starts with a sharp decline of the storage modulus at low temperatures, followed by a more gradual loss of connectivity at elevated temperatures. The gels formed in the presence of salts have higher moduli at temperatures above 35 °C. This suggests very different macroscopic structures of fibril networks in samples without and with salt in line with the above microscopy results in the aforementioned section indicated. The biphasic behavior is attributed to the two sets of fibrils observed in the no salt microscopy image indicated in Fig. 2. The homogenous network produced by the addition of salt stabilizes the gel at lower temperatures. Interestingly, the G′ values at room temperature obtained for the employed salts are only moderately different for the cations (Mg^2+^  > NH_4_^+^ > Na^+^, which does not follow Hofmeister) and practically identical for the employed anions. This observation is clearly different from findings with Fmoc-VL and Fmoc-LL peptides^13^.

The gel → sol temperatures, *T*_*gs*_, in Fig. 3b were obtained using the point at which G″ > G′. Obviously, all the employed salts decrease *T*_*gs*_. The hierarchy of *T*_*gs*_ values of GHG gels prepared with different cations are consistent with the corresponding Hofmeister series in that they decrease from kosmotropes to chaotropes by 9 °C, while the *T*_*gs*_ values observed with different anions are identical within the limits of experimental accuracy. This is a surprising result, since the influence of Hofmeister anions on protein and fibril stability is generally more pronounced^22^.

In addition to *T*_*gs*_ we determined the softening point from the complex modulus, G^*^^23^ (Supplementary Fig. S5). This softening point reflects a loss of percolation or fibril connectivity. The obtained softening temperatures do not indicate any ion specificity in that all values lie between 40.9 and 42.0 °C except for NH_4_^+^ (45.9 °C). Thus, we can conclude that regarding the gel softening the influence of NH_4_^+^ is negligible.

### UV-CD spectroscopy

**Figure 4:**
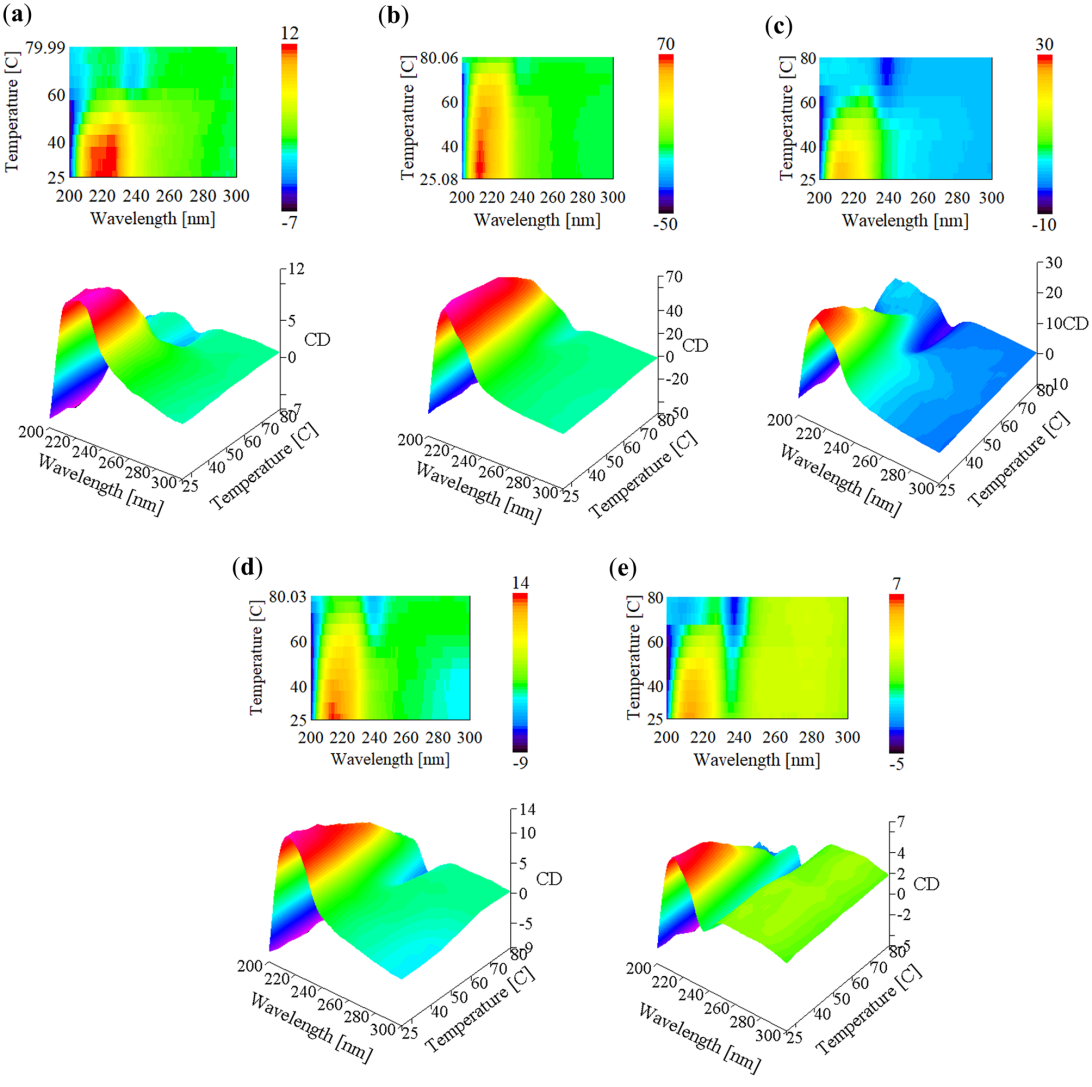
Thermal dissolution of GHG fibrils probed by UV circular dichroism. 3D thermal and 2D heat plots of the UVCD spectra of the GHG gel formed in the presence (**a**) 0.1 M NH_4_Cl, (**b**) no added salt, (**c**) 0.1 M MgCl_2_, (**d**) 0.1 M NaCl, and (**e**) 0.1 M NaCH_3_COO. The fibril system is stable across most temperatures with a decrease in the ECD signal appearing only at higher temperatures. The sodium nitrate sample produced a significantly nosier spectra compared to other samples.

UV-circular dichroism was used to probe the interactions that lead to the self-assembly and specifics of the individual fibril structure. Figure 4a–e exhibits 3D-plots of the UVCD spectra of a gel produced with 80 mM GHG in the absence and presence of additional salt measured at different temperatures and spectra measured in the presence of ions. They show exactly the predicted behavior in that the dichroism decreases over a broad wavelength region. The spectrum of a GHG monomer measured at different pH are shown for comparison (Supplementary Fig. S6)^2^. The deprotonation of the imidazole side chain produces a spectrum with a very broad and weak positive maximum between 190 and 220 nm.

**Figure 5:**
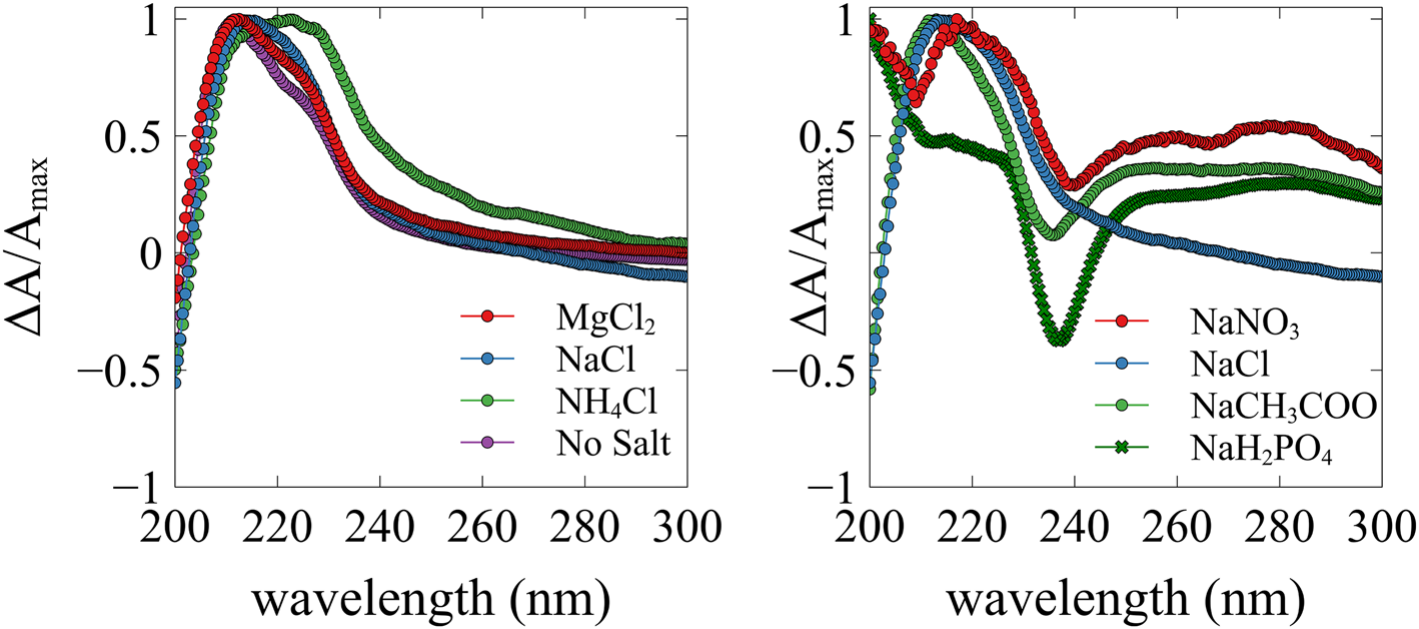
Normalized UV-CD spectra for gels made with various cations (left) and anions (right) probed at room temperature. The sodium nitrate sample produced a significantly nosier spectra compared to other samples.

The room temperature gel spectra in Fig. 5 are qualitatively and quantitatively different from the monomer spectrum of the zwitterionic state. The spectra of the gel formed in the presence of NaNO_3_ was too noisy for monitoring it over the temperature interval and is therefore omitted. We normalized the spectra, since absolute values for the molar dichroism were difficult to obtain because the effective concentration of peptides in the optical path depends on the sample preparation. Therefore, we just focus on relative changes in this study. Most of spectra in Fig. 4 depict a broad and intense positive maximum at 215 nm, which decays as a function of increasing temperature. Contrary to spectra of secondary structures of polypeptides the spectrum exhibits considerable rotational strength in the region between 230 and 245 nm, which is clearly diagnostic of a Psi-CD type enhancement (cf. the Supporting Information Discussion D1 for a brief explanation of the underlying physics)^24^.

While the spectra of the ‘no salt’ sample and the ones of the cationic series are qualitatively similar, those of the anionic series are clearly different at 240 nm, where they display a pronounced negative maximum, Fig. 5 compares spectra at room temperature. The intensities of the negative maxima scale with the Hofmeister series for non-chloride anions, i.e. PO_4_^2−^ > CHCOO^−^ > NO_3_^−^ (from salting out to salting in). This observation indicates that the listed anions change the fibrillar structure in a way which most likely affects the ππ-stacking between imidazole rings to which rotational strength in this region could be attributed, because it frequently produces a bathochromic shift of the absorption spectrum transitions^25^. Since the room temperature G′-values of the anion series are nearly identical, the CD-detected structural changes have no impact on the connectivity in the gel phase. It should also be noted in this context that broad positive rotational strength above 240 nm is likely to originate from a dichroism of light scattering^24^.

At high temperatures the spectrum resembles the one of zwitterionic monomers overlapped by a pronounced negative maximum at 240 nm which is now present in all spectra. The existence of a negative maximum in the high temperature spectrum, whereby the fibrils are almost completely dissolved, suggests that some ππ-stacking in soluble peptide oligomers still exists and that it resembles the one observed for different anions at room temperature.

**Figure 6:**
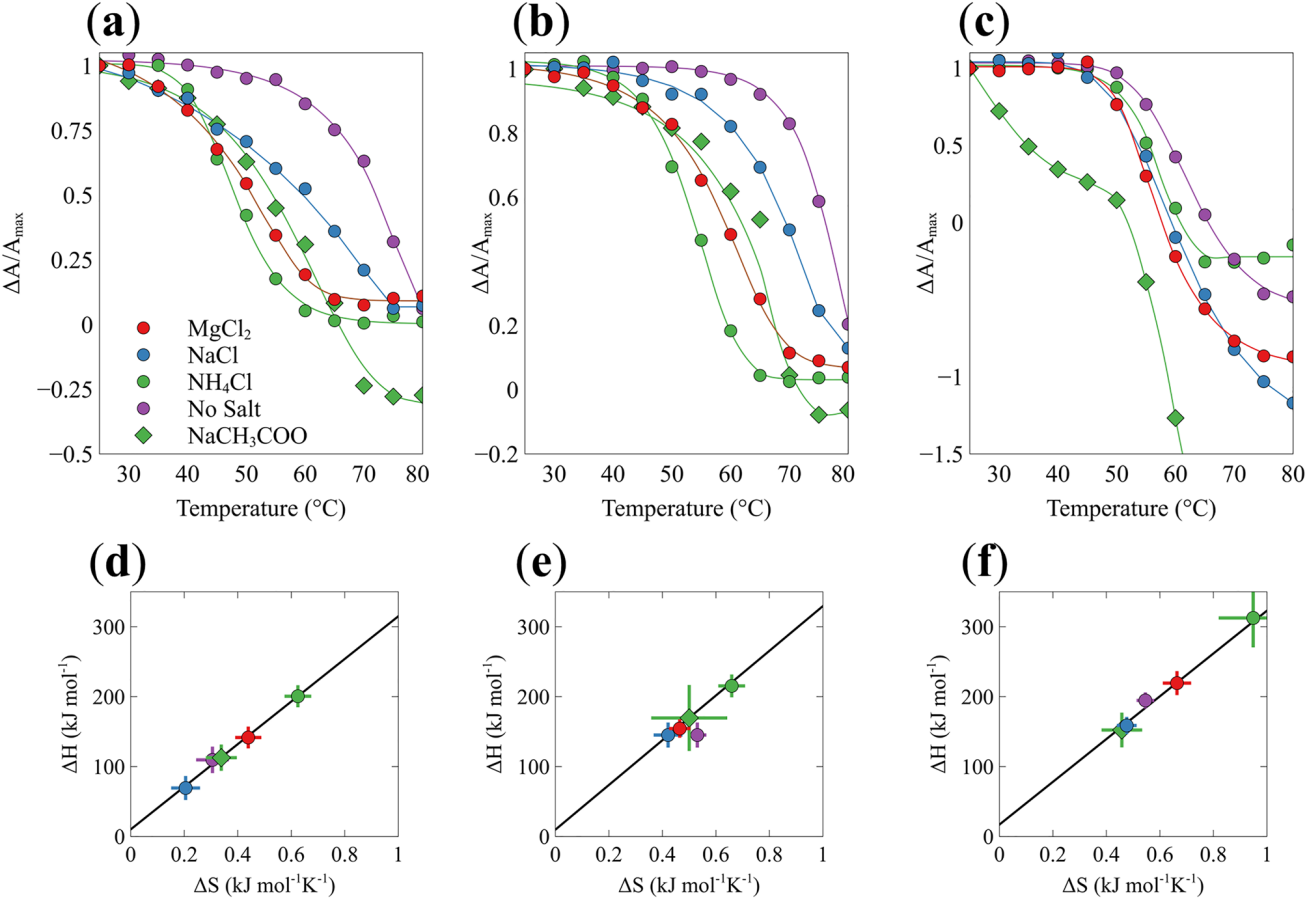
GHG fibril dissolution probed by UVCD spectroscopy. The sigmoidal dissolution curves for (**a**) 214 nm, (**b**) 228 nm, and (**c**) 240 nm of the GHG systems are fitted with of a two-state model curve relating parameters to thermodynamic state functions. The CD was normalized using the positive maximum value at each wavelength to enable comparison of curves. Correlation plot of the thermodynamic parameters $\Delta {H}_{sf}$ and $\Delta {S}_{sf}$ obtained from changes of dichroism values at wavelengths (**d**) 214 nm, (**e**) 228 nm, and (**f**) 240 nm.

For a further analysis of the CD-dissolution curves we plotted the change in the dichroism at three wavelengths (214, 228 and 240 nm) as a function of temperature in Fig. 6a–c. For the sake of comparison all dissolution curves were normalized on the dichroism value obtained at the lowest temperature.

In a first step we analyzed the dissolution curves in Fig. 6a–c in terms of the two-state model, in line with the approach of Farrell et al.^26^ outlined in Supplementary Discussion D2. These states should not be confused with the existence of only two species. The two thermodynamic states should be rather envisaged as ensembles of fibrils of different size and solubilized peptides in different aggregation states (monomers included), respectively. If the dissolution of the fibrils produced a homogeneous ensemble of peptides (only monomers or the same type of oligomers), the thermodynamic parameters obtained from the fits to the dissolution curves constructed with the dichroism values at different wavelengths would be identical within the limits of statistical uncertainty. However, the thermodynamic parameter values listed in Supplementary Tables S1–S3 reveal significant differences. Supplementary Discussion D3 addresses this issue, here we focus on parameter values obtained at 240 nm that predominantly probes the interaction of $\pi \pi$-interactions of the histidine side chain.

The increase in enthalpy destabilization of the gel state is nearly neutralized by an entropic gain. To corroborate this view, we plotted *ΔH*_*sf*_ versus the corresponding entropy *ΔS*_*sf*_ in Fig. 6d–f.

The plots shown in Fig. 6 are indicative of a linear enthalpy-entropy compensation. We subjected them to a linear regression based on the equation^27^:

3 $$\Delta {H}_{sf}=\Delta {H}_{sf}^{0}+{T}_{c}\Delta {S}_{sf}$$

where *T*_*c*_ is the compensation temperature and $\Delta {H}_{sf}^{0}$ denotes the excess enthalpy. The compensation temperature is expected to be close to the transition temperature at which total enthalpic and entropic compensation is obtained, i.e. $\Delta {G}_{sf}=0$, if the uncompensated excess enthalpy is not too large (Table 1 lists the linear fitting parameters obtained from the enthalpy-entropy plots). The correlation parameters for all three fits are close to 1 indicating a strong linear correlation between enthalpy and entropy. The obtained compensation temperatures are all below the dissolution temperatures. This is due to the small but not insignificant excess (i.e., not compensated) enthalpy that stabilizes the gel phase and that is not affected by the choice of the salt (Supplementary Fig. S7). A larger fraction of the dissolution enthalpy and entropy is just varied by the addition of salt in a way which involves total enthalpy-entropy compensation at T_c_.

**Table 1:** Fitting parameters for $\Delta {{H}}_{{sf}}/\Delta {S}_{sf}$ plots.

	214 nm	228 nm	240 nm
T_d_ (K)	304.85 ± 13	306.06 ± 36	319.91 ± 17
$\Delta {H}_{sf}^{0}(\frac{{\text{kJ}}}{\text{mol}})$	10.109 ± 6	16.952 ± 19	9.7027 ± 11
R^2^	0.9987	0.9605	0.9917

A comparison of softening, gel → sol transition dissolution temperature of the fibrils (Supplementary Tables S1–S3) reveals another aspect of the salt influence. In the absence of the latter, the dissolution temperatures are slightly higher than the respective gel → sol temperatures. This suggests that the dissolution of the gel phase (loss of connectivity, i.e. percolation) precedes the dissolution of the fibrils, in line with what was earlier observed for GAG in water/ethanol^26^. In the presence of salt, however, the dissolution temperatures are slightly lower than the gel → sol temperatures, which indicates that the loss of the gel status is preceded by the dissolution of fibrils. The softening temperatures are the lowest of all three. That makes sense because they mark the onset of the loss of connectivity.

## Discussion

### Two phases versus monophase melting

The rheology-based gel → sol curve for GHG in the absence of salt shows a biphasic behavior with two softening points around 29 °C and then again at 69 °C. The second phase leads to the complete solubilization of the gel network, while the first one produces a significant decrease of the gel strength. In the presence of salt, the softening becomes monophasic with G′ values below the ‘no-salt’ values at room and above in an intermediate temperature region. We propose that the presence of ions “slows” the formation of fibrils thus promoting the growth of homogeneous clusters formed by long fibrils all the while simultaneously hindering the number of nucleation sites, most likely due to either a dilution effect of the condensed phase or increasing the solubility of the peptide. This leads to larger voids which reduces the connectivity and thus the strength of the gel network. Figure 2 shows clusters in these gels shrink in length first and then disappear. In the absence of salt, nucleation sites are abundant and promote the growth of a heterogeneous mixture of fibrils. We attribute the different stabilities of the two sub-ensembles to differences in the density of each type of fibril cluster. The concentration of monomers and soluble aggregates reaches saturation in the crowded vicinity of smaller fibrils early which prevents further dissolution. This inhibitory effect is absent for longer fibrils. This rationale explains why the dissolution temperature of the fibrils is lower than the gel → sol transition temperature in the absence of salt, while it is the other way round in the presence of salt.

### Influence of cations and anions

The obtained temperature parameters all suggest that the addition of salt reduces the stability of gel phase and fibrils. This is a non-specific effect that reflects the dependence of the transition temperature on the ionic strength of the solvent. The specific influence of the added salts on the melting temperature of the gel suggests a weak cationic Hofmeister effect while no specific trend is observed with the changing of anions. The chaotropic ion Mg^2+^ caused the greatest thermal destabilization of the gel with *T*_*gs*_ lowered by 10 °C. It is very likely that this destabilization is due to the strong coordination of Mg^2+^ ions by the C-terminal carboxylate group of the peptide^7^. We have recently shown that the x-direction of the unit cell is colinear with the long axis of the fibril (cf. the crystal structure in Supplementary Fig. S8). Thus, the unique surfaces of the fibril are amphiphilic in nature with one of the xz-surfaces being hydrophilic and riddled with both ammonium and carboxylate sites, while the opposing xz-surface is slightly more hydrophobic due to the exposed imidazole rings. The carboxylate groups are closer to the surface and should produce an attractive potential for ions. Besides an accumulation of cations in the hydration shell the ions can form ion pairs with the carboxylate groups. On the contrary, the anions would have to overcome a potential barrier prior to pairing with the ammonium group. Cations in the hydration shell of hydrophilic site of fibrils could inhibit the sticking between different fibrils by prevent imidazole groups from the more hydrophobic site of another fibril to form hydrogen bonds with COO^−^ and from getting involved in anion-π interactions. That would destabilize the gel state. Generally, preferential binding of cations to carboxylate group would stabilize soluble peptides which offers much more binding sites.

### UV circular dichroism

It can be expected that the CD-signal is size-dependent until the dimension of a fibril reaches a certain threshold. For the UVCD of α-helices the threshold lies at ca. 20 residues which would correspond to a distance of 10.8 nm^28^. For fibrils like the ones formed by GHG the threshold might be dictated by the wavelength of the absorbed light which would be still far below the length and the widths of GHG fibrils. Hence, if fibrils dissolve predominantly via the dissociation of monomers or small fragments the rotational strength indicated by the UVCD spectrum would substantially decrease with increasing temperature to reflect the increasing concentration of dissolved peptides that stay monomeric or cluster into amorphous aggregates^29^.

### Fibril length and CD

While the rheological gel → sol transition curves of GHG without salt is biphasic the corresponding dissolution curves obtained from the temperature dependence of UV dichroism values are clearly monophasic with dissolution temperatures close but not identical with the respective gel → sol transition temperatures. On first view this is a surprising result, since the images shown in Fig. 2 suggest that the loss of connectivity and the dissolution of fibrils are somewhat intertwined. The only possible explanation for this observation is that the fragments that are formed by the dissolution of the long fibrils are of a size that exceeds the threshold value for changes of the CD spectrum (vide supra). As a consequence, changes of the CD spectrum only occur when the shorter fibrils dissolve into soluble monomers and oligomers to a size which lies below the threshold value. Supplementary Figure S9 shows that as the GHG fibril network/connectivity decreases, a reduction in the CD intensity is observed. The sample contains much less visible fibrils and most likely smaller aggregates.

### Enthalpy–entropy compensation

The nearly total enthalpy–entropy compensation is noteworthy in that the uncompensated enthalpy is comparatively small though still sufficient to produce a compensation temperature that is below the dissolution temperature. The interested reader can find a qualitative discussion in the SI (Supplementary Discussion D3). Here we just mention that the observed compensation most likely obfuscates any ion specificity. It is obvious from our data that individual enthalpy and entropy values are actually ion specific but they do not show a Hofmeister behavior.

## Conclusion

In the absence of salt GHG forms a heterogeneous set of fibrils that originate from droplet like condensates formed via liquid–liquid phase separation. Rheology reveals a biphasic gel → sol transition. The addition of different salts increases the solubility of the peptide which leads to lower transition temperatures. Investigating different cations and anions of the Hofmeister series revealed that contrary to expectation the ion specificity regarding the observed decrease of the gel → sol temperature follows the Hofmeister hierarchy for the cations. For anions, we observed differences between the CD-spectra but no significant influence on the gel → sol temperature. We attribute these findings to specific interactions of cations with COO^-^ groups on the surface of the fibrils. The addition of ions homogenizes the gel by reducing the number of condensates. That facilitates a more homogeneous growth of fibrils. Thus, the corresponding gel → sol transitions become monophasic.

The dissolution of fibrils was probed by UVCD measurements as a function of temperature. All dissolution curves are monophasic and could be analyzed in terms of a model, which assumes that the long fibrils are in thermodynamic equilibrium with the sol phase in which some GHG peptides are still incorporated in soluble aggregates. While the investigated ions affect the enthalpy and entropy of dissolution in different ways (the most pronounced change was observed for Mg^2+^ and NH_4_^+^), they reduce the dissolution temperature of fibrils by nearly the same amount due to a nearly total enthalpy-entropy compensation. The ion specific changes of thermodynamic parameters do not show a Hofmeister behavior. The obtained data suggest the gel phase would still be thermodynamically stable at physiological temperatures.

The calculated solubility and softening temperature for GHG are higher compared to other investigated GXG peptides^14^. Stabilization of the GHG fibrils outlined in this paper could be due to the hydrogen bonding capacity of the imidazole which can function as both a donor and acceptor. The possible influence of side chain involving hydrogen bonding on the dissolution temperature of fibrils is also apparent for MAX peptides, where the substitution of valine by threonine leads to a much higher dissolution temperature^30^. Compared with the results of studies on other short peptides the influence of cations and anions on the stability of GHG fibrils and the gel phase is moderate^7,13^.

### Outlook

The set of Hofmeister ions utilized in this study is of course incomplete. We did not investigate cations and anions are the edge of current Hofmeister scales (guanidinium, thiocyanate and SO_4_^−^ ions). The use of guanidinium chloride could be of particular interest in view of its capability to induce protein denaturation. Moreover, one may ask whether the combination of different Hofmeister ions could produce some new synergetic effects which would be worth investigating.

Observational studies currently suggest the dissolution of GHG gels in the presence of an aqueous medium is dependent on the presence of ions. At low volumes (< 1 mL) salt gels dissolve at a faster rate compared to the gels made with no salts. This is in line with the observation that the presence of salt produces greater voids in the hydrogel material, and it is most likely the higher surface area available to bulk water that facilitates the faster dissolution. This needs to be confirmed with more quantitative studies. Currently we are investigating the dissolution of GHG gels using frequency shifts assignable to the aromatic protons of the histidine side chain in nuclear magnetic resonance spectra along with more thermal rheology studies.

## Materials and methods

### Preparation of 80 mM GHG gel samples

Peptide powder was purchased from Bachem with a guaranteed removal of trifluoroacetic acid. The hydrogels were prepared by the addition of deionized water or a 0.1 M salt solution and heated to above 80 °C until the peptide fully dissolves. The peptide gel is formed once removed from the heating source and allowed to cool to room temperature. Hydrogels were prepared with 80 mM peptide concentration. This value lies close to the critical aggregation concentration (CAC), which is approximately 40–50 mM for GHG. Staying close to the CAC threshold reduces the ionic strength from the peptide’s salt (HCl). Moreover, a higher concentration would impede the quality of the CD spectra owing to the strong absorptivity of the protein in the 200 nm region.

### Small amplitude oscillatory shear rheology

Rheology measurements were obtained on a DHR-3 rheometer (TA instruments) using a Peltier plate for temperature control with a 25 mm diameter top plate. For each measurement, we used 400 µL of peptide solution and a gap size of 700 µm. A solvent trap was devised using safflower oil around the free surface edge of the sample to minimize evaporation (see Supplementary Fig. S11). Samples were prepared and formed on the rheometer. Gelation was probed by measuring the storage modulus G′ versus time over a period of 5000 s by applying a strain of 0.03%, and an angular frequency of 1 rad/s at 20 °C. Subsequently, the storage and loss modulus of the sample was measured as a function of temperature after sample equilibration by using a temperature ramp of 1 °C/min between 25 and 90 °C.

### Ultra-violet electronic circular dichroism

Spectra were collected on a Jasco J-1200 spectropolarimeter purged with nitrogen. The samples were loaded into a 200 μm cell and sealed with silicone based vacuum grease to prevent evaporation. UV-CD spectra were recorded between 190 and 300 nm using a scan speed of 500 nm min^−1^, a data pitch of 0.05 nm, and a band width of 5 nm. Five accumulations were averaged for each time interval and a D.I.T. of 1 s. For each measurement, the system was first incubated at different initial temperatures for different time periods to ensure that equilibrium was established (example shown in Supplementary Fig. S10).

### Bright field microscopy

Samples were initially allowed to form on a microscope slide with a coverslip. Images were collected using an Amscope BH200-MR Series Metallurgical Microscope.

### Supplementary Information

Supplementary Information.

## Data Availability

The authors will make their original data available upon reasonable request.
