# Supplementary material for: Tuning the thermostability of GHG gels by salts at different positions on the Hofmeister scale
Source: Sci Rep. 2024 Jun 26;14:14742. doi: 10.1038/s41598-024-65145-7 (PMC11208536; doi:10.1038/s41598-024-65145-7)
Supplement: Supplementary file 1 — Supplementary Information. [file 41598_2024_65145_MOESM1_ESM.pdf]

Tuning the Thermostability of GHG Gels by Salts at Different Positions on the Hofmeister Scale.

Nichole S. O'Neill[1], Nicolas J. Alvarez[2]* and Reinhard Schweitzer-Stenner[1]**

[1]Department of Chemistry and [2]Department of Chemical and Biological Engineering, Drexel University, Philadelphia, PA 19104, USA

Submitted to: *Scientific Reports*

*Corresponding authors, email: nja49@drexel.edu and rs344@drexel.edu

Table of Contents

Supplemental Figures

Figure S1. Formation of GHG/0.1 M NaCl Gel

Figure S2. Formation of GHG (No Added Salt) Gel

Figure S3. Rheology Repeated Measurements

Figure S4. Rheology Storage and Loss Moduli

Figure S5. Rheology Softening Temperature

Figure S6. UV-CD Spectrum GHG Monomer

Figure S7. UV-CD Enthalpy-Entropy Analysis

Figure S8. GHG Fibril Crystalline Structure

Figure S9. UV-CD Anisotropy Analysis

Figure S10. UV-CD Steady State Experiment

Figure S11. Rheometer Solvent Trap

Supplemental Tables

Table S1. Thermodynamic Parameters from Fitting of UV-CD Curves at 214 nm

Table S2. Thermodynamic Parameters from Fitting of UV-CD Curves at 228 nm

Table S3. Thermodynamic Parameters from Fitting of UV-CD Curves at 240 nm

Supplemental Details

Discussion D1. Psi-type Dichroism

Discussion D2. Two-state Thermodynamics Model

Discussion D3. Inhomogeneity of ECD Thermodynamic Parameters

Discussion D4. Enthalpy-Entropy Compensation

Supplemental Figures

Figure S1. Still images of the formation of a GHG hydrogel made in the presence of 0.1 M NaCl. The time lapse between photo A1 and D3 is approximately 27 minutes. The lengths observed in the images are on the mm scale (the rheometer plate has a radius of 12.5 mm).

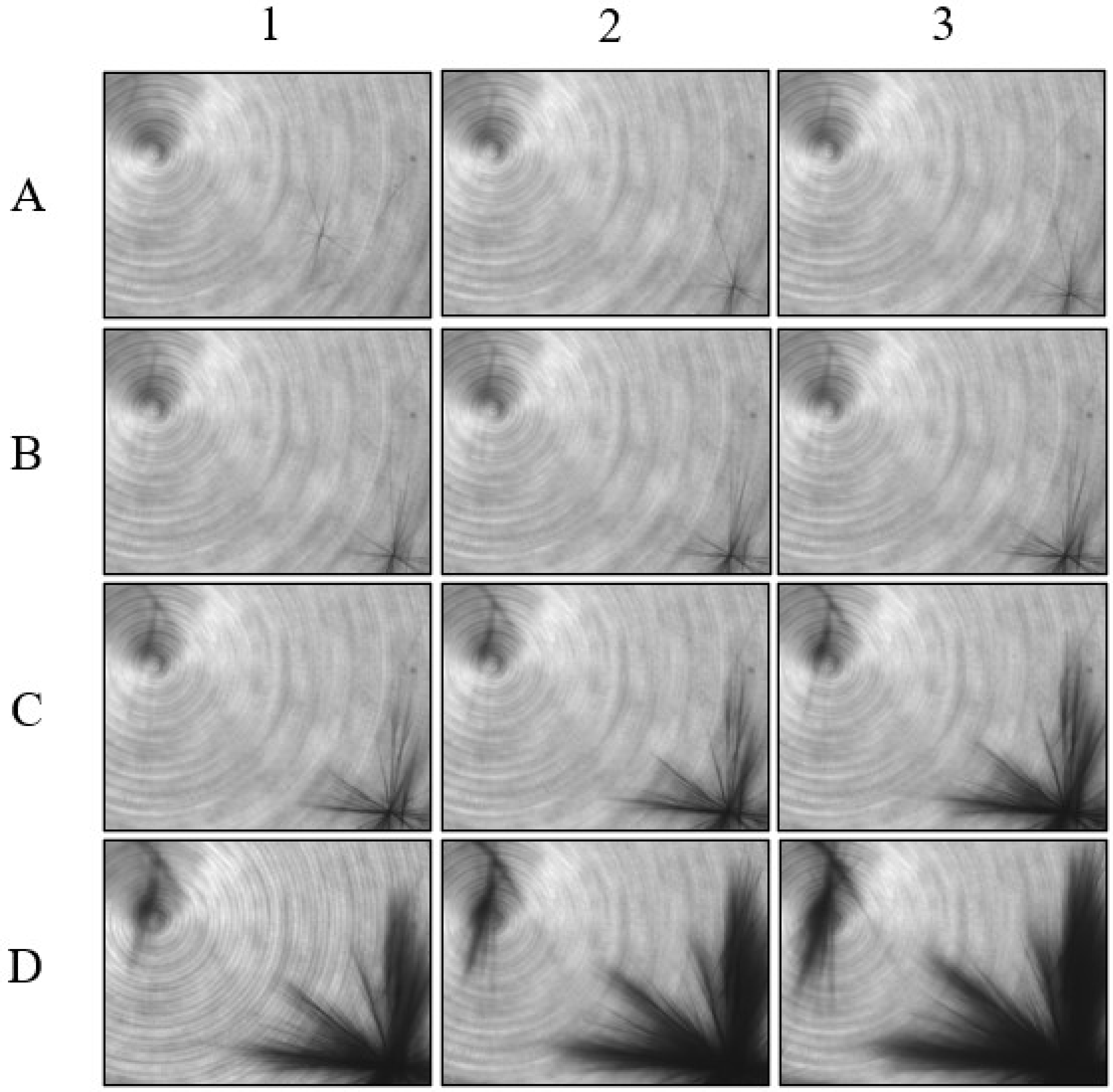

Figure S2. Still images of the gel → sol transition of a GHG hydrogel made in the absence of additional salt (low ionic strength). The temperature interval between photo A1 and D3 is approximately 67°C to 73°C. The lengths observed in the images are on the mm scale (the rheometer plate has a radius of 12.5 mm).

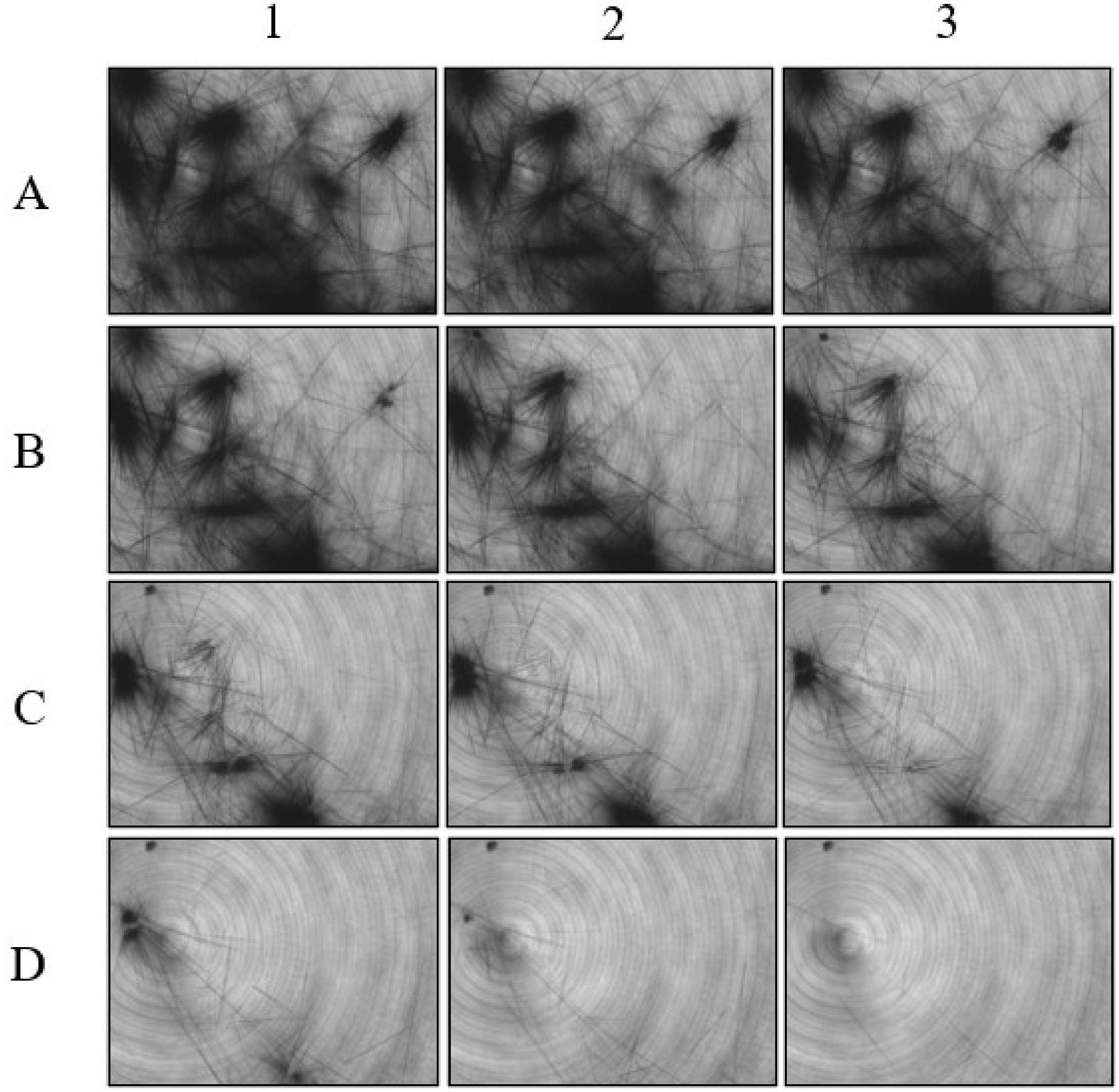

Figure S3. Triplicate measurements of $MgCl_2$ and NH_4Cl were performed to obtain an average storage and loss modulus as a function of temperature. The average T_{gs} values for NH_4Cl and $MgCl_2$ were calculated as 71.3±2.1°C and 62.3±0.96°C, respectively. All samples formed a gel network, as indicated by storage moduli in the 10^4 Pa range at room temperature.

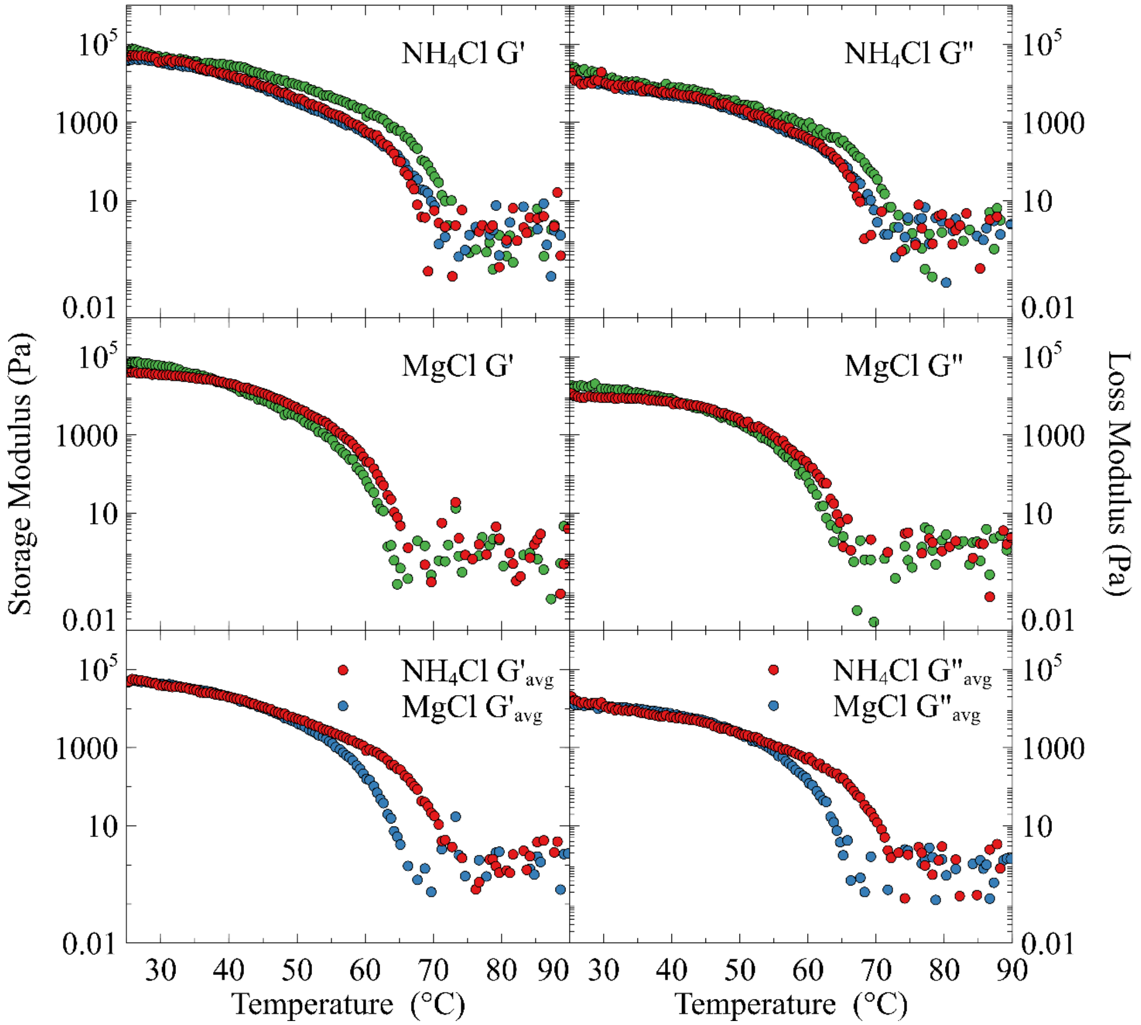

Figure S4. The storage (top) and loss (bottom) moduli for all gels studied are compare below. The tanδ was also calculated for room temperature 25 °C and is presented below the rheology curves. There is no observable trend in the tanδ values outside of salt versus no salt.

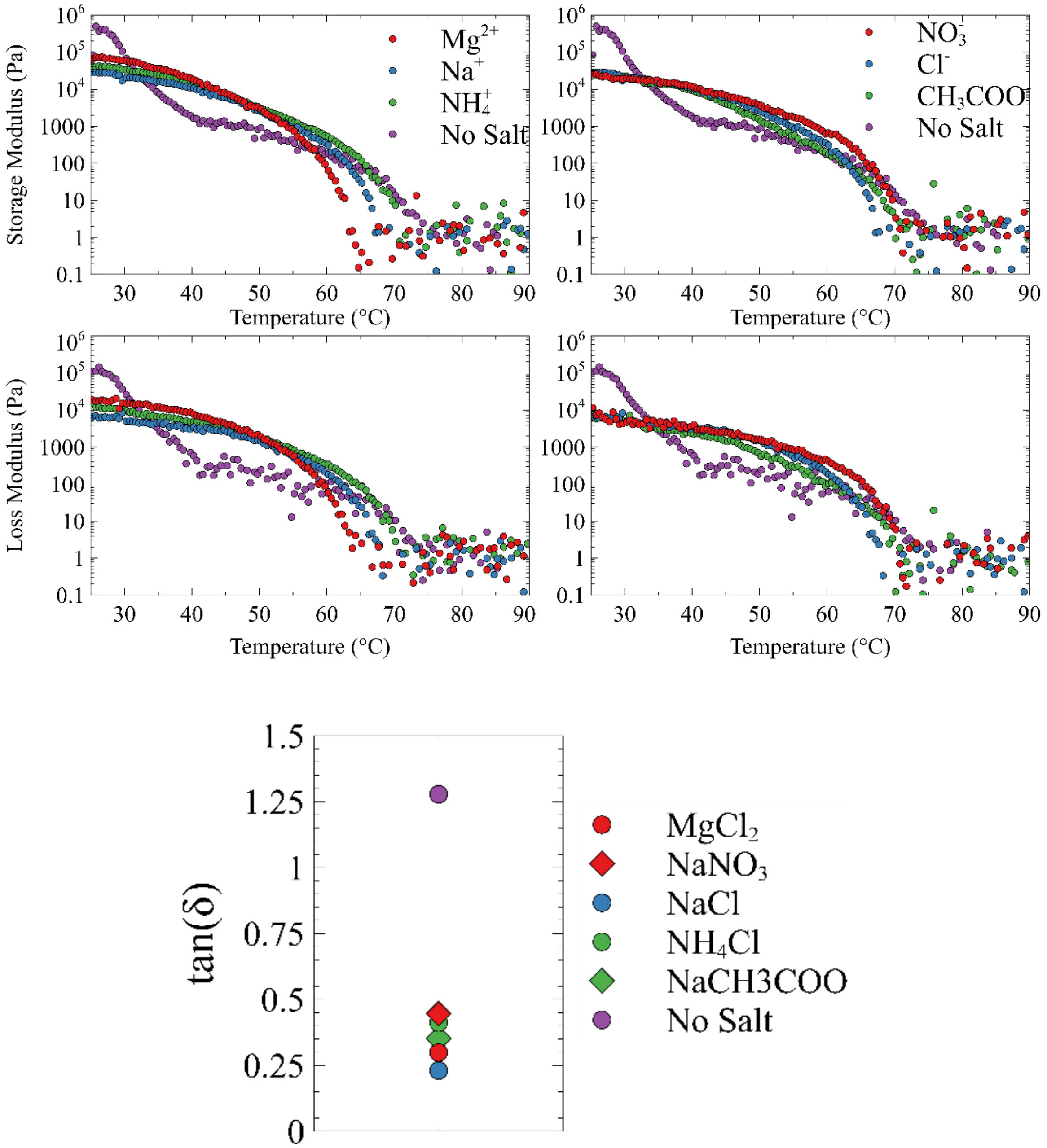

Figure S5. (a) The melting curves were characterized by graphically determining a softening point which coincides with the observed "knee." This value was determined using the initial modulus "plateau" as well as the first region of sharp modulus decrease. These regions were fitted using the LINEST function in Excel and (**b**) the $T_{soft,\ gel}$ value was recorded as the point of intersection.

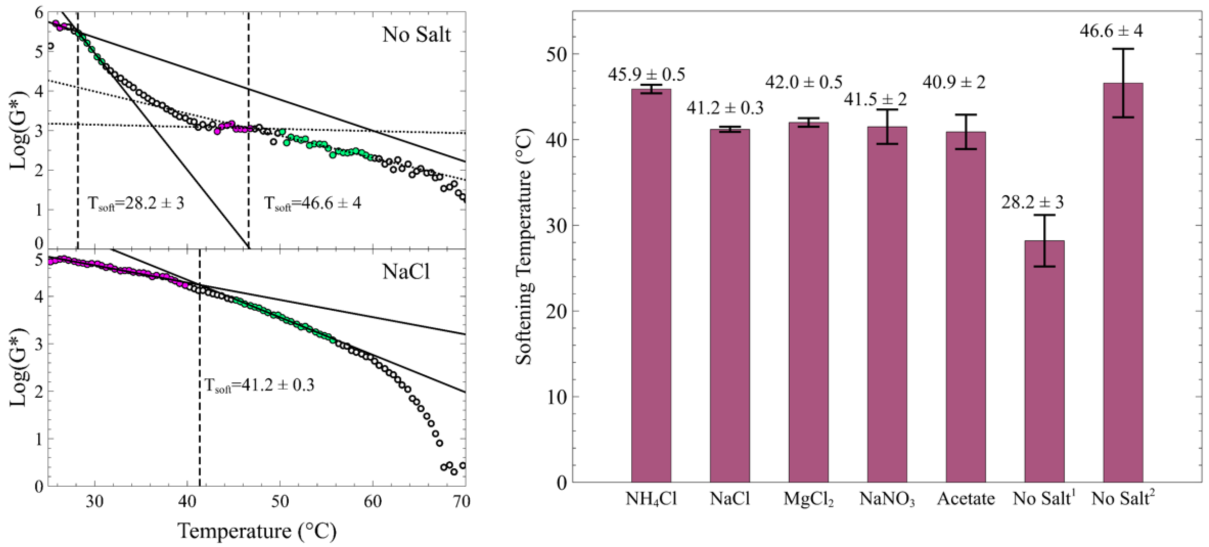

Figure S6. UV-CD spectra of a 10 mM monomeric GHG sample in water was recorded at the indicated pH-values in a region between 2.8 and 10.6. The spectra display an isodichroic point. Figure adapted from ref.[1]

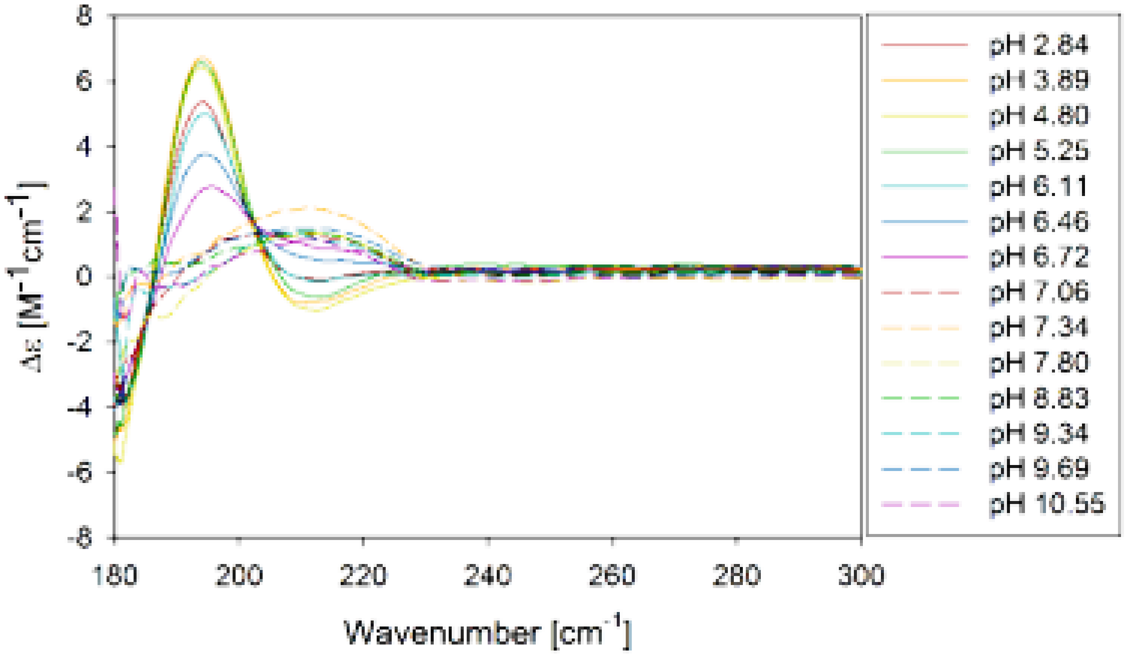

Figure S7. Comparison of the enthalpy-entropy values obtained by the analysis of UVCD data collected at wavelengths 214 nm (top), 228 nm (middle), and 240 nm (bottom).

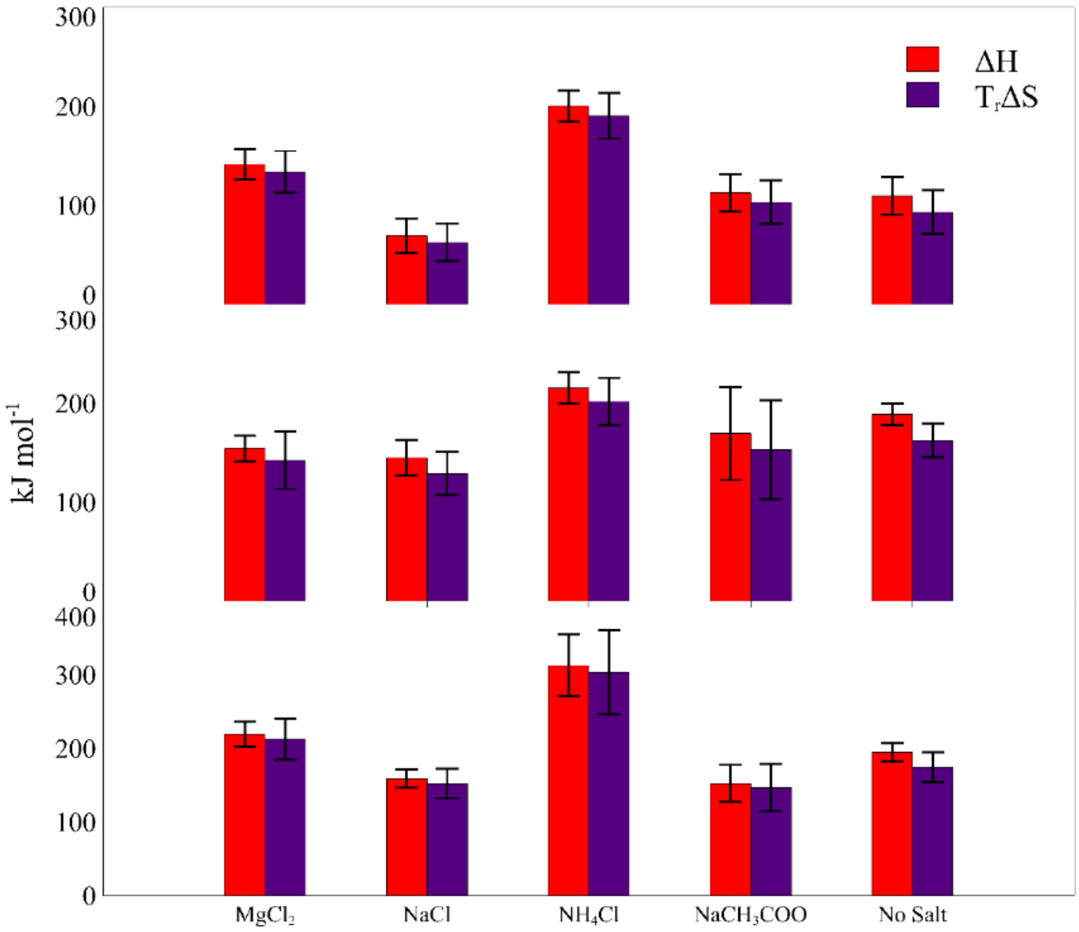

Figure S8: Nanostructure of GHG fibrils are amphilic containing hydrophobic and hydrophilic surfaces. The long axis is decorated with carboxylate groups which are thought to contribute the effects observed with the cation salt series.

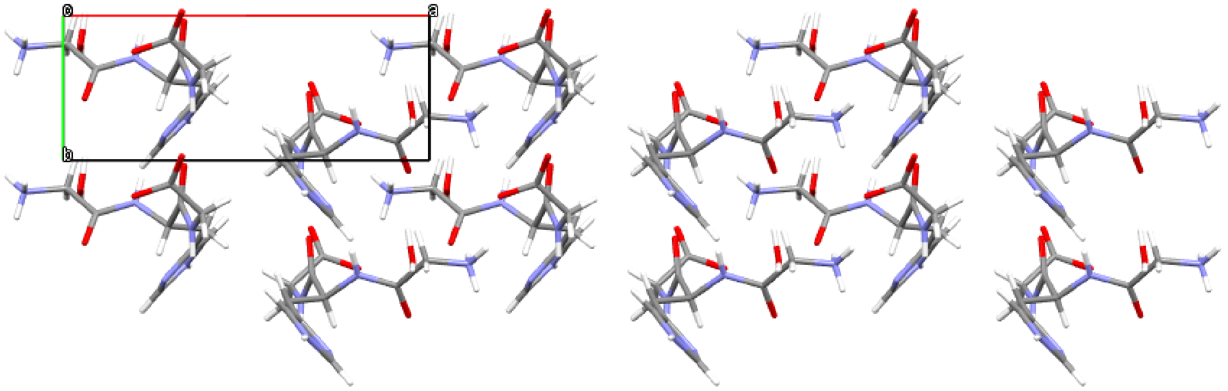

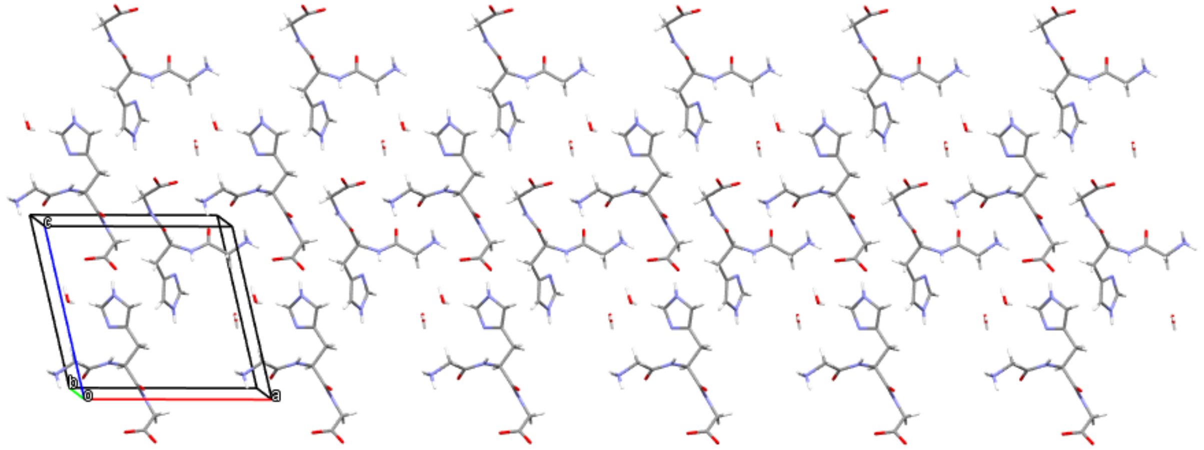

Figure S9. ECD samples measured at different angles and with a visible difference in crosslinking/fragment sizes. The top spectra are replicate measurements of 80 mM GHG samples made with no salt measured at the standard position (R0) and a 180° out-of-plane rotation. Samples were plated with an excess volume and then sealed with silicone grease. The middle and bottom spectra were plated with smaller volumes which lead to a difference in visible crossing-linking.

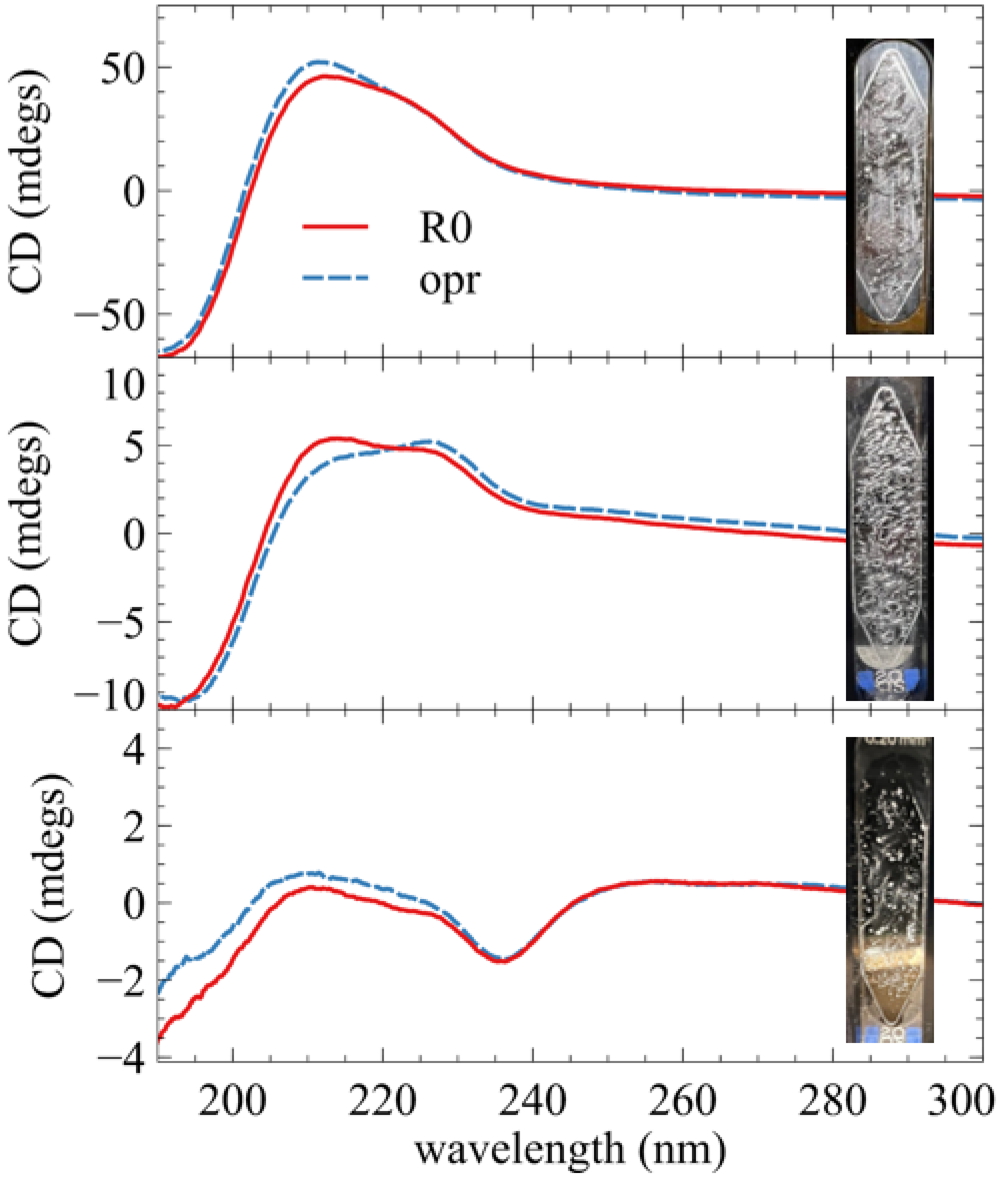

Figure S10. GHG gels were monitored using UV-CD to ensure equilibrium was achieved between switching temperature intervals. A steady state was achieved after 5 minutes at each temperature.

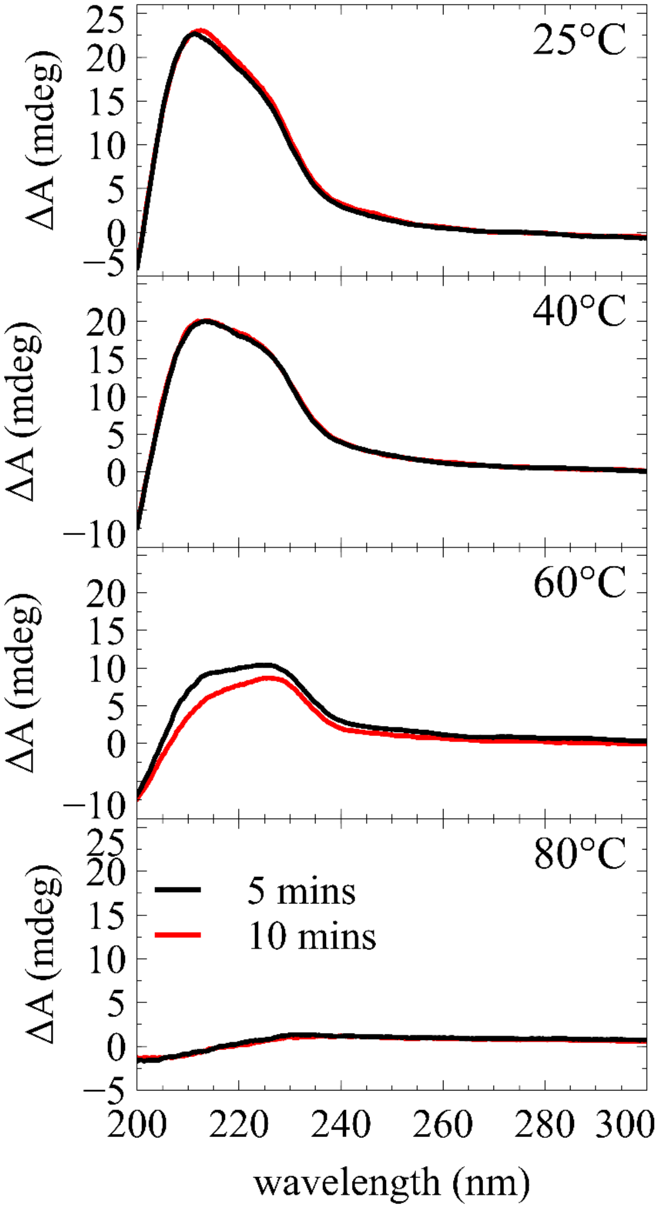

Figure S11. The melting temperatures of the gels reported herein are well below the boiling temperature of the solvent (water). Evaporation due to the thermal heating between the two plates is not possible. However, the evaporation at the edges could occur. To prevent this the sample is surrounded with excess safflower oil which we have previously validated does not interfere with the instrument measurement nor causes slipping of the plates.

The image below shows the heigh difference between the gel material and the top plate of the DHR-3 instrument. A wall of puddy entraps the safflower oil which is used to prevent evaporation from the edges (oil not shown). The oil height is ¼ to ½ the height of the top plate. None of the microscopy images of the formation/melting process indicate evaporation at the boundaries or interference from the safflower oil.

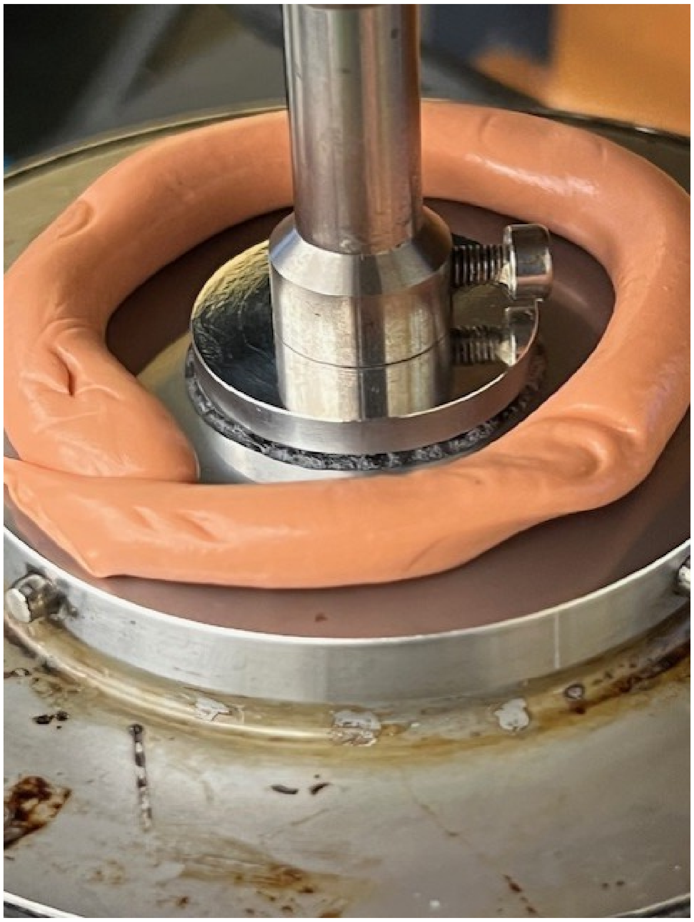

Supplemental Tables

Table S1. Thermodynamic Parameters from Fitting of UV-CD Curves at 214 nm

**System**	**Δεgel $(Mcm)^{-1}$**	**Δεsol $(Mcm)^{-1}$**	**ΔH (kJ/mol)**	**T_d(K)**
0.1M $MgCl_2$	1.0112 ± 0.0265	0.0639 ± 0.0225	141.6 ± 15.6	322.3 ± 0.7
0.1M NaCl	1.0366 ± 0.0572	-0.0320 ± 0.2547	69.33 ± 17.2	337.9 ± 5.0
0.1M NH_4Cl	1.0186 ± 0.0173	0.0065 ± 0.0135	200.6 ± 15.8	321.1 ± 0.4
0.1M $NaCH_3COO$	0.9772 ± 0.0415	-0.4583 ± 0.1055	112.7 ± 18.8	332.9 ± 1.5
No Salt	1.017 ± 0.0143	-1.6659 ± 1.4948	109.6 ± 18.9	358.7 ± 9.2

Table S2. Thermodynamic Parameters from Fitting of UV-CD Curves at 228 nm

**System**	**Δεgel $(Mcm)^{-1}$**	**Δεsol $(Mcm)^{-1}$**	**ΔH (kJ/mol)**	**T_d(K)**
0.1M $MgCl_2$	0.9859 ± 0.0134	0.0171 ± 0.0268	154.4 ± 13.1	332.1 ± 0.6
0.1M NaCl	0.9993 ± 0.0118	-0.01379 ± 0.1159	145.1 ± 17.9	344.4 ± 1.6
0.1M NH_4Cl	1.0110 ± 0.0132	0.0144 ± 0.0151	215.4 ± 16.4	326.8 ± 0.4
0.1M $NaCH_3COO$	0.2192 ± 0.1470	0.9421 ± 0.0380	169.5 ± 47.2	338.8 ± 2.1
No Salt	0.9994 ± 0.0027	-1.111 ± 0.3982	188.8 ± 11.3	355.9 ± 1.8

Table S3. Thermodynamic Parameters from Fitting of UV-CD Curves at 240 nm

**System**	**Δεgel $(Mcm)^{-1}$**	**Δεsol $(Mcm)^{-1}$**	**ΔH (kJ/mol)**	**T_d(K)**
0.1M $MgCl_2$	1.0238 ± 0.0230	-0.8719 ± 0.0337	219.4 ± 17.2	330.5 ± 0.4
0.1M NaCl	1.0665 ± 0.0268	-1.2341 ± 0.0611	158.8 ± 12.2	333.6 ± 0.5
0.1M NH_4Cl	1.006 ± 0.0228	-0.2330 ± 0.0287	312.6 ± 42.1	329.5 ± 0.4
0.1M $NaCH_3COO$	0.2386 ± 0.0340	-1.0659 ± 0.0723	152.3 ± 24.8	332.7 ± 1.1
No Salt	1.0420 ± 0.126	-0.5434 ± 0.0304	194.6 ± 11.5	356.6 ± 0.3

Supplemental Discussion

Discussion D1. Psi-type Dichroism

The observed rotational strength (dichroism value) is produced by electronic coupling between $\pi \rightarrow \pi^*$ and $n \rightarrow \pi^*$ transitions of the intrinsically achiral aromatic side chains and peptide groups, both causing a delocalization of excited electronic states.[2–7] Hence, the obtained UVCD spectrum of the investigated gels reflect the chirality of the underlying crystal fibrils rather than the one of individual peptide monomers in the unit cell. For secondary structure fragments of proteins, the delocalization is generally explained with a coupled oscillator model that invokes static transition dipole coupling between the above oscillators[7,8]. However, in supramolecular structures this model is insufficient because the classical dipole approximation is no longer valid[9]. Radiative coupling via oscillating dipoles and so-called intermediate coupling terms reflects contributions to the electronic states that lead to significant enhancement of CD spectra in the region of optical absorption (Psi-type dichroism) which is diagnostic of a much more pronounced delocalization of electronic states[10].

Discussion D2. Two-state Thermodynamics Model

The apparent δΔA/A values were thus described by:

$$\delta\Delta|\mathrm{A}|(T) = \frac{\delta\Delta|\mathrm{A}|_{fib} + \delta\Delta|\mathrm{A}|_{sol}\cdot\exp\left(\frac{-\Delta H_{sf}}{RT} + \frac{\Delta S_{sf}}{R}\right)}{1 + \exp\left(\frac{-\Delta H_{sf}}{RT} + \frac{\Delta S_{sf}}{R}\right)} \quad \text{(S1)}$$

$$|A| = \frac{A}{A_{max}} \quad \text{(S2)}$$

Eq. S1 was fitted to the data with the Dynamic Fit Wizard in Sigma Plot which allowed us to characterize the melting of the gel in thermodynamic terms as previously done for GAG gels[10]. $\delta\Delta|\mathrm{A}|_{\mathrm{gel}}$ and $\delta\Delta|\mathrm{A}|_{\mathrm{sol}}$ are the differential dichroism values for the fibril and the solution phase, respectively, for three wavelength (214 nm, 228 nm, or 240 nm). $\Delta\mathrm{H}_{\mathrm{sf}} = H_{sol} - H_{fib}$ and $\Delta\mathrm{S}_{\mathrm{sf}} = S_{sol} - S_{fib}$ denote the dissolution enthalpy and entropy, respectively. *R* is the gas constant and *T* is the absolute temperature. Instead of the dissolution entropy we used the dissolution temperature $T_d = \Delta H_{sf}/\Delta S_{sf}$ as a free parameter (see Tables S1-S3). Here, we ignore possible changes of heat capacity which produce temperature dependent enthalpies and entropies.

Discussion D3. Inhomogeneity of ECD Thermodynamic Parameters

While the differences between respective T_d values are modest, the value for the thermodynamic parameters ΔH_{sf} and ΔS_{sf} vary considerably. Our results thus show that the dichroism values taken at the three wavelengths probe the formation of different sub-ensembles of a heterogeneous dissolution process. Generally, the species probed at 228 and 240 nm seem to be more enthalpically disfavored than the ones that contributes to the dichroism at 214 nm. A discussion of these observations is given below.

Spectral inhomogeneity. Our two-state analysis of the CD-data yielded different enthalpy and entropy values for data sets taken at different wavelengths in the broad wavelength range of the CD spectrum. This observation can be qualitatively understood when we first consider the high probability that ππ-stacking causes a bathochromic shift of the electronic transitions of the imidazole side chain.[12] The latter can be expected to lead to an extension of the already broad imidazole spectrum towards longer wavelengths. It is therefore reasonable to assume that the CD-values taken at 240 nm solely reflect the dissociation of ππ-stacking. The negative rotational strength observed at high temperatures indicates that some coupling is left but that it now reflects a different ππ-interaction in soluble oligomers. All the other wavelengths probe a combination of changes involving interpeptide hydrogen bonding and ππ-stacking.

In order to explain our observations, we invoke a scenario in which the ensemble of solubilized peptides populated at higher temperatures is highly heterogeneous. Depending on the extent of hydrogen bonding, the intrinsic backbone conformation of the peptides, imidazole tautomerization and different involvements of ππ-stacking the corresponding CD spectra can be expected to be different. Moreover, it is well thinkable that the dissolution process is also thermodynamically heterogeneous. If dissolution predominantly involves the dissociation of monomers the dissociation process might be followed by aggregations associated with different formation enthalpies and entropies. The more enthalpically favored the soluble aggregates are, the more do they reduce the apparent disfavorable enthalpy of the dissolution process. By the same token, they reduce the entropy gain.

It should be noted that the three T_d values of the 'no salt' sample are practically identical. In the presence of NaCl and $MgCl_2$ the melting temperature derived from the 224 nm data is slightly higher than the one obtained from the 214 and 240 nm data set. The rather modest differences between corresponding melting temperatures contrast with the significant variations of melting enthalpies and entropies. These observations suggest that the latter lie all close to the same enthalpy-entropy correlation line, in agreement with the rather modest differences between T_c and ΔH_{sg}^0 values.

One might wonder whether the discrepancies between the parameters obtained for the dichroism change at different wavelengths might be due to turbidity. Due to the size of formed fibrils none on the investigated gel samples was transparent. Turbidity causes an increase of the baseline in the absorption spectrum. However, as shown by Farrell et al. for GAG gels formed in ethanol-water, the turbidity has only a very small influence on the measured dichroism in the region of the absorption band[10]. This is in line with the theoretical calculations of Keller and Bustamante[13] Anisotropic effects were also considered; however, rejected after measuring the CD at for different orientations of the sample in the spectrometer. Both a 180° in-plane and out-of-plane rotation resulted in minimal changes in the relative intensities suggesting no spectral features are assignable to either birefringence or scattering anomalies.

Discussion D4. Enthalpy-Entropy Compensation

The total enthalpy-entropy balance can be written as follows:

$$\Delta G_{sf} = \Delta H_{sf,c} - \mathrm{T}(\Delta \mathrm{S}_{sf,c} - \Delta S_{sf,t}^{p} - \Delta S_{sf,t}^{s} - \Delta S_{sf,r}^{p} - \Delta S_{sf,r}^{s}) + \Delta H_{sf,hb} - T\Delta S_{sf,hb} + \Delta H_{sf,hyd} - T\Delta S_{sf,hyd} + \Delta H_{sf,a} - T\Delta S_{sf,a} + \Delta H_{sf,salt} - T\Delta S_{sf,salt} + \Delta H_{sf,rel} - T\Delta S_{sf,rel} \quad (3)$$

where the subscripts and superscripts have the following meaning: *c* means conformational, *t* translational, *r* rotational, *hb* H-bonding, *hyd* hydrophobic, *a* aromatic, *salt* saltbridge and *rel* relaxation. The conformation enthalpy differs from ΔH_c^0 in that it contains a hydration part. The conformational entropy

change reflects solely the reduction of Ramachandran plot sampling in the fibril state and the solvation induced change of the vibrational entropy. The conformation change is unfavorable for the monomeric peptide in water. Since the unit cell structure lies half-way between the polyproline II and β-strand conformation the overall enthalpic change might be minimal since these equally populated regions are enthalpically favored and disfavored, respectively[14]. However, the reduction of the conformational distribution comes with an entropic cost[15]. The entropic penalty caused by the elimination of translation and rotational entropy of the peptides in the gel phase can be expected to be large[16]. However, these losses are most likely compensated by the entropy gains of the water molecules that are squeezed out of the fibrils[17]. Several lines of evidence suggest that water release might be a substantial driving force governing the formation of amyloid fibrils. The desolvation of aliphatic and aromatic groups (CH_2) are generally believed to be entropy driven[18]. The formation of very strong hydrogen bonds should produce negative values for $\Delta H_{sf,hb}$ at partially neutralized by a decrease in vibrational entropy[19]. The thermodynamic balance of the salt bridges is somewhat unclear. Several lines of evidence suggest that salt bridge formation in proteins is entropy driven (release of water), while the enthalpy is unfavorable because of the large negative solvation energy of the involved groups.[20] However, the distances between two groups suggest very strong interactions (ca. 2Å for H---O distances, between 2.7 and 2.9 Å for N—O distances, the corresponding cut off values are 3 and 4Å, respectively). Hence, it is well likely, that their formation is enthalpy driven while the strong bonding reduces the entropy to an extent that exceeds the gain produced by water release. The last two terms are difficult to assess. They represent the relaxation of water molecules caused by creating a cage into which the reactants (peptide monomers) and the products (fibrils) are inserted from vacuum. In view of individual $\Delta H_{sf,rel}$ and $\Delta S_{sf,rel}$ values might be large and even overshadow the other contributions to the Gibbs energy[21]. However, as shown theoretically, they can be expected to exhibit total enthalpy-entropy compensation at all temperatures[22]. Even though we cannot quantify individual contributions at the present time, the above line of argument makes the observed enthalpy-entropy compensation very plausible.

The absence of any Hofmeister-type ion specific influence of the ionic strength on the dissolution temperature could be due to enthalpy-entropy compensation. The obtained compensation is not too far away from ideal, in which case one would observe total compensation at the transition temperature (*Td* = *Tc*). In such case different salts just affect enthalpy and entropy while the result Gibbs energy contribution would be small in the vicinity of the dissolution temperature. The current understanding of the uncompensated enthalpy ΔH_0 is that it represents the enthalpy of a conformational change *in vacuo*. Naturally, for a very short peptide like GxG this value would be much smaller than the one of e.g. Aβ, prion protein fragments or even proteins like tau, alpha-synuclein, etc. Since $T_d=\Delta H_0/\Delta S_{fs}+Tc$, the influence of different cations/anions on ΔS shift T_d, if ΔH0 is sufficiently large. This does not seem to be the case for GHG fibrils.

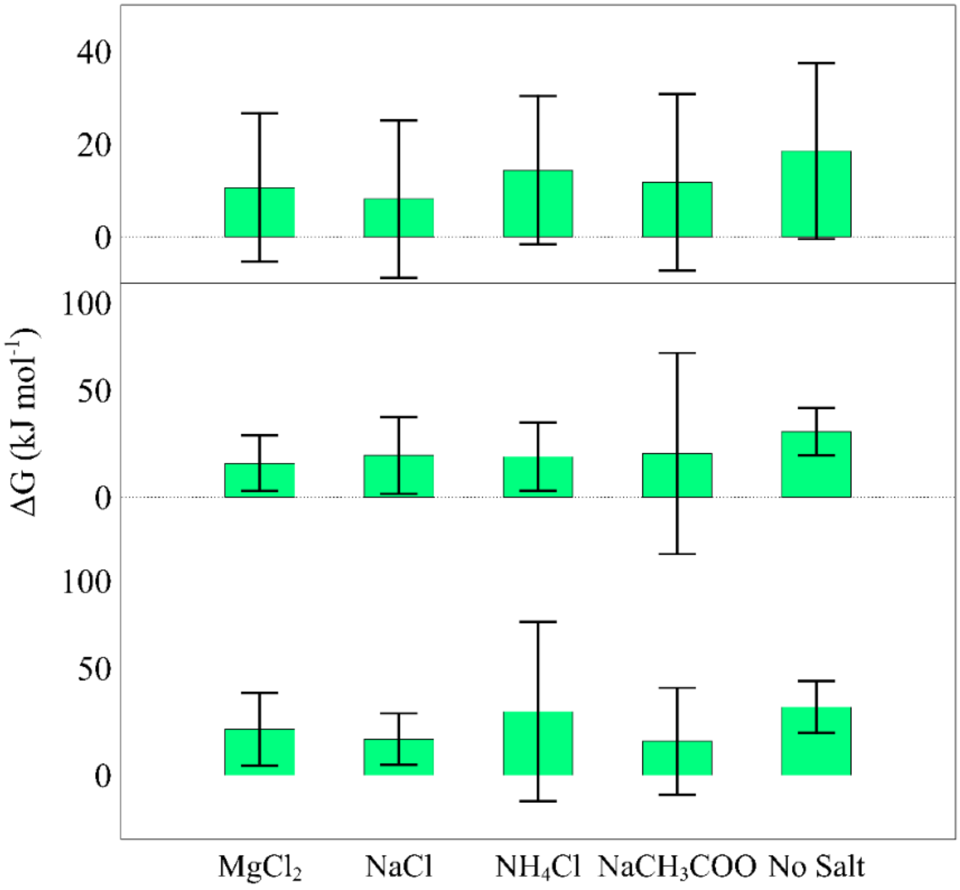

References

1. Hesser, M. *et al.* The tripeptide GHG as an unexpected hydrogelator triggered by imidazole deprotonation. *Soft Matter* **16**, 4110–4114 (2020).

2. Tinoco, I. Theoretical Aspects of Optical Activity Part Two: Polymers. *Adv. Chem. Phys.* **4**, 113–160 (2007).

3. Woody, R. W. Aromatic side-chain contributions to the far ultraviolet circular dichroism of peptides and proteins. *Biopolymers* **17**, 1451–1467 (1978).

4. Manning, M. C. & Woody, R. W. Theoretical Study of the Contribution of Aromatic Side Chains to the Circular Dichroism of Basic Bovine Pancreatic Trypsin Inhibitor. *Biochemistry* **28**, 8609–8613 (1989).

5. Hirst, J. D., Colella, K. & Gilbert, A. T. B. Electronic circular dichroism of proteins from first-principles calculations. *Journal of Physical Chemistry B* **107**, 11813–11819 (2003).

6. Bhattacharjee, S., Tóth, G., Lovas, S. & Hirst, J. D. Influence of tyrosine on the electronic circular dichroism of helical peptides. *Journal of Physical Chemistry B* **107**, 8682–8688 (2003).

7. Sreerama, N. & Woody, R. W. Computation and Analysis of Protein Circular Dichroism Spectra. in 318–351 (2004). doi:10.1016/S0076-6879(04)83013-1.

8. Woody, R. W. & Tinoco, I. Optical Rotation of Oriented Helices. III. Calculation of the Rotatory Dispersion and Circular Dichroism of the Alpha- and 310-Helix. *J Chem Phys* **46**, 4927–4945 (1967).

9. Keller, D. & Bustamante, C. Theory of the interaction of light with large inhomogeneous molecular aggregates. I. Absorption. *J Chem Phys* **84**, 2961–2971 (1986).

10. Farrell, S., DiGuiseppi, D., Alvarez, N. & Schweitzer-Stenner, R. The interplay of aggregation, fibrillization and gelation of an unexpected low molecular weight gelator: Glycylalanylglycine in ethanol/water. *Soft Matter* **12**, 6096–6110 (2016).

11. Koren, A. B., Curtis, M. D., Francis, A. H. & Kampf, J. W. Intermolecular Interactions in π-Stacked Conjugated Molecules. Synthesis, Structure, and Spectral Characterization of Alkyl Bithiazole Oligomers. *J Am Chem Soc* **125**, 5040–5050 (2003).

12. Keller, D. & Bustamante, C. Theory of the interaction of light with large inhomogeneous molecular aggregates. II. Psi-type circular dichroism. *J Chem Phys* **84**, 2972–2980 (1986).

13. DiGuiseppi, D. & Schweitzer-Stenner, R. Probing conformational propensities of histidine in different protonation states of the unblocked glycyl-histidyl-glycine peptide by vibrational and NMR spectroscopy. *Journal of Raman Spectroscopy* **47**, 1063–1072 (2016).

14. Schweitzer-Stenner, R. & Toal, S. E. Entropy reduction in unfolded peptides (and proteins) due to conformational preferences of amino acid residues. *Physical Chemistry Chemical Physics* **16**, 22527–22536 (2014).

15. Workman, R. J. & Pettitt, B. M. Thermodynamic Compensation in Peptides Following Liquid–Liquid Phase Separation. *J Phys Chem B* **125**, 6431–6439 (2021).

16. Thirumalai, D., Reddy, G. & Straub, J. E. Role of water in protein aggregation and amyloid polymorphism. *Acc Chem Res* **45**, 83–92 (2012).

17. Makhatadze, G. I. & Privalov, P. L. Energetics of Protein Structure. in 307–425 (1995). doi:10.1016/S0065-3233(08)60548-3.

18. Dunitz, J. D. Win some, lose some: enthalpy-entropy compensation in weak intermolecular interactions. *Chem Biol* **2**, 709–712 (1995).

19. Lee, H., Dehez, F., Chipot, C., Lim, H.-K. & Kim, H. Enthalpy–Entropy Interplay in π-Stacking Interaction of Benzene Dimer in Water. *J Chem Theory Comput* **15**, 1538–1545 (2019).

20. Ben-Naim, A. *Molecular Theory of Water and Aqueous Solutions. Part II: The Role of Water in Protein Folding, Self-Assembly and Molecular Recognition*. (World Scientific Publishing, Singapore, 2011).

21. Grunwald, E. & Steel, C. Solvent Reorganization and Thermodynamic Enthalpy-Entropy Compensation. *J. Am. Chem. Soc* **117**, 5687–5692 (1995).
